# Seasonal assessment of groundwater quality and irrigation suitability using a solar-powered AgNP/activated carbon treatment system in a coastal aquifer

**DOI:** 10.1038/s41598-026-66183-z

**Published:** 2026-08-24

**Authors:** Mohamed Y. Omar, Rania A. Ibrahim, Ebtesam El Bestawy, Kareem M. Tonbol, Ragaa A. Ahmed

**Affiliations:** 1 Deanery of Scientific Research and Innovation, Arab Academy for Science, Technology and Maritime Transport, Alexandria, 21937 Egypt; 2 Electrical and Control Engineering Department, College of Engineering and Technology, Arab Academy for Science and Technology, Alexandria, 21937 Egypt; 3 Department of Environmental Studies, Institute of Graduate Studies and Research, Alexandria University, 832 Alexandria, Egypt; 4 Maritime Post Graduate Studies Institute, Arab Academy for Science, Technology & Maritime Transport, Alexandria, 21937 Egypt

**Keywords:** Environmental sciences, Hydrology

## Abstract

The increasing water scarcity, groundwater degradation, and climate variability have increased the demand for sustainable groundwater management strategies in arid and semiarid regions. This work presents an integrated framework for groundwater monitoring and irrigation suitability assessment of groundwater from 13 borehole at a university campus in Alexandria, Egypt, under climate change conditions. The developed system was implemented via a field-scale, solar-powered fixed-bed column packed with a green-synthesized silver-nanoparticle/activated-carbon nanocomposite (AgNPs/AC-NC). Groundwater from the 13 wells was sampled over 4 consecutive seasons to investigate the spatial and temporal variations in physicochemical, microbiological, and hydrochemical characteristics before and after treatment. In addition, water quality indices were calculated to assess water suitability for irrigation in accordance with the Food and Agriculture Organization of the United Nations (FAO) guidelines. The preliminary hydrochemical analysis of the raw samples indicated that sodium and bicarbonate ions were the dominant ions, which is typical for the sodium-bicarbonate water type, with elevated salinity, turbidity and dissolved constituents during the warm seasons. The AgNPs/AC-NC treatment resulted in reductions in salinity-related parameters, major dissolved ions, and irrigation hazard indices averaged across all wells and seasons. Most of the physicochemical parameters showed an overall reduction of 30–80%, with the most significant reductions in sulphate (80.3%), alkalinity (71.0%), sodium and potassium (67.4%), nitrate (52.6%), electrical conductivity (54.2%), chloride (53.5%), and total dissolved solids (47.3%). In addition, the potential salinity, residual sodium carbonate and total hardness decreased by 50–72%, and the total and fecal coliform concentrations were < 1 CFU/100 mL in most samples. While most of the parameters were within the FAO irrigation standards, copper, nitrite, acidity and turbidity remained above the standard, suggesting that a dedicated copper removal and polishing step is still needed. Nonetheless, the framework provides a practical, decentralized groundwater treatment and irrigation reuse model that is climate-resilient, making it suitable in water-scarce regions.

## Introduction

A reliable and adequate water supply is crucial for human development and survival. In countries with limited freshwater resources, concerns over water safety for drinking and irrigation have posed significant challenges to long-term socioeconomic growth. Like many other countries, Egypt suffers from serious water supply problems arising from high demand and the depletion of conventional resources. In accordance with Egypt’s national development plans for the period 2037–2050 for various sectors, an annual water deficit of 21 billion cubic meters (BCM) is expected with continuous growth^1^, and the per capita water availability has declined to approximately 570 m³/year, which is well below the international water scarcity threshold of 1,000 m³^2^. Moreover, owing to the inherent uncertainty of the Egyptian water resource system and the heavy reliance on the Nile River to supply nearly 90% of the total freshwater supply, major challenges include the rapidly widening gap between limited water availability and the increasing demand for freshwater unless other nonconventional sources are developed and integrated within a sustainable water management framework^3,4^.

Groundwater has consequently become an increasingly important supplementary resource in Egypt^5^, particularly for agriculture, which represents the largest water-consuming sector in many regions and uses approximately 37.1 billion cubic meters annually for irrigation^6^. However, owing to the increased stress on groundwater resources, the intrusion of saline water and contamination from fertilizers and untreated wastewater have degraded their quantity and quality in several aquifers. These physical and chemical deteriorations reduce the suitability of groundwater for irrigation, harm crop productivity, and may result in additional health risks if not properly assessed, monitored or treated^7^. Accordingly, recent research has increasingly examined renewable-energy-powered groundwater abstraction and treatment as a means of reducing operating costs and carbon emissions. Recent studies in Egypt have emphasized this need by specifying suitable zones for solar-powered groundwater exploitation through technoeconomic and multicriteria analyses^8,9^. In addition, optimizing groundwater pumping to avoid over-exploitation of stressed aquifers has been explored in^10^. Moreover, optimizing the performance of a solar-powered submersible pump system using artificial intelligence^11^ and digital twins enables more sustainable water management solutions in resource-constrained environments for long-term irrigation^12^. Beyond the Egyptian context, many studies have addressed decentralized, renewable-energy-powered water treatment. Solar-powered reverse osmosis (RO) has consolidated the technoeconomic and operational basis for using photovoltaic solar power to supply membrane processes in regions characterized by high solar irradiance and freshwater scarcity^13^, whereas wider syntheses have examined the integration of solar energy with decentralized treatment and disinfection technologies as a combined response to water stress and energy constraints^14^. At the implementation level, field and pilot studies have demonstrated the feasibility of off-grid, solar-powered operation in rural Kenya^15^ and a fully autonomous, pilot-scale system in an arid region of Mexico with integrated photovoltaic supply, RO, and solar photocatalysis with real-time monitoring, cost-aware decentralized operation^16,17^.

Following these challenges, numerous studies have emphasized the importance of systematic groundwater quality assessment and management. Water quality indices (WQIs) have been extensively used to combine physicochemical parameters into a single numerical value that reflects overall water suitability for drinking and irrigation purposes^18^. With respect to irrigation use, several irrigation indices, such as SAR, residual sodium carbonate (RSC), PI, PS, and magnesium hazard, exist^19^. On the basis of these assessment approaches, a range of treatment and management strategies have been proposed to improve water quality and alleviate pressure on conventional freshwater resources.

To date, there are various methods for treating groundwater for irrigation purposes, especially for saline and brackish water, in arid and semiarid regions such as Egypt. Among these, adsorption-based treatment technologies have been widely adopted due to their simple operation, flexibility, and effectiveness in enhancing the quality of groundwater^20^. Commonly, adsorption-based treatments often use granular activated carbon, which has the ability to remove organic compounds, color, odor, and some inorganic contaminants through physical and chemical interactions at the solid‒liquid interface. However, in recent years, significant progress has been made in materials science, resulting in the development of nan-oenabled water treatment technologies, which use nanomaterials to modify traditional adsorbents to increase surface area and reactivity^21^. Recent reviews have established that nanomaterials can act simultaneously as adsorbents, catalysts, and antimicrobial agents owing to their high specific surface area and reactivity, making them a versatile platform for removing both conventional and emerging contaminants^22–26^. Metal nanoparticles, especially silver nanoparticles, have gained popularity because of their high antimicrobial activity and ability to enhance contaminant removal when they are immobilized on carbon-based materials^27^. As a result, adsorption‒disinfection hybrid technologies have been developed as integrated technologies that combine the contaminant adsorption ability of activated carbon with the antimicrobial properties of nanoscale materials^28^. Such silver-on-carbon systems are most accurately described as metal-nanoparticle/carbon nanocomposites, in which the carbon provides an adsorptive surface while the immobilized silver delivers disinfection within a single medium^29^. However, the application of AgNPs/activated carbon nanocomposites may be limited by possible silver leaching, reduced efficiency after repeated reuse, and sensitivity to groundwater chemistry, such as salinity, pH, and competing ions. Therefore, long-term stability, regeneration performance, and environmental safety should be further evaluated before large-scale field application^30^. Other approaches, such as desalination-based methods^31–33^, membrane-based technologies^34–36^, electrodialysis^37,38^, and electrochemical methods^39,40^, have also been investigated. However, these techniques are often associated with high capital and operating costs, substantial energy requirements, and membrane fouling, which may limit their suitability for decentralized irrigation applications.

The selection of AgNPs/AC-NC rather than activated carbon or silver nanoparticles alone is based on the complementary functions of the two materials. Activated carbon provides a high-surface-area porous matrix for the adsorption of organic and inorganic pollutants but has limited intrinsic antimicrobial activity^41^. Silver nanoparticles provide strong antimicrobial activity and high surface reactivity^42,43^. Their immobilization on activated carbon therefore creates a multifunctional medium that combines contaminant adsorption with microbial reduction while reducing the direct use and dispersion of unsupported nanoparticles^44,45^.

Despite the numerous studies covering groundwater studies in Egypt, most existing research focuses on assessment, mapping, modelling, or risk classification, whereas fewer studies focus on field-based treatment and verified reuse. In particular, limited focus has been allocated to integrated systems that combine seasonal groundwater monitoring, irrigation suitability evaluation, and renewable-energy-powered onsite treatment within a single operational framework. This gap is particularly relevant for coastal and semiurban areas, where groundwater quality is influenced by seasonal variability, salinity- related constraints, dissolved constituents and microbiological contamination. Hence, there is a need for decentralized and climate-resilient groundwater reuse systems that not only diagnose water quality deterioration but also offer a practical treatment route for irrigation applications.

This research proposes an integrated system for groundwater monitoring, irrigation suitability evaluation, and solar-powered nanocomposite treatment to improve groundwater suitability for irrigation applications. The developed framework was implemented and experimentally evaluated at the AASTMT campus in Alexandria, Egypt, under real operational conditions for 13 boreholes. The main contributions of this paper can be summarized as follows:

- An integrated groundwater management framework is presented, combining seasonal groundwater monitoring, hydrochemical assessment, irrigation suitability evaluation, solar-powered abstraction, and AgNPs/AC-NC treatment within a single operational platform, whereas previous Egyptian studies have typically treated groundwater assessment and treatment as separate problems.
- While AgNPs/AC-NC have been used in Egypt in previous studies for drinking water disinfection and wastewater or heavy metal removal, in this work, the material is applied to groundwater for irrigation reuse, which is evaluated against a comprehensive suite of irrigation suitability indices across four seasons.
- Unlike current groundwater treatment studies in Egypt, which are generally limited to laboratory studies or single-stage treatment processes, this paper presents a decentralized nanocomposite filtration system at the field scale that has been experimentally implemented and validated under real operation conditions for sustainable irrigation water reuse.
- By integrating seasonal characterization, irrigation water-quality indices, renewable-energy-driven operation, and nano-enabled treatment in a single platform, the framework offers a realistic and climate-resilient model for groundwater reuse under evolving environmental conditions.

The rest of the paper is organized into nine sections. Section 2 describes the recent literature covering existing studies related to groundwater in Egypt, highlights the existing research gaps and emphasizes the contributions of this paper. Section 3 describes the study area and the distribution of groundwater wells on the AASTMT campus, Alexandria, Egypt. The proposed methodology including groundwater sampling and analysis, design of the solar powered-filtration system and performance evaluation indices are described in Sect. 4. Section 5 describes the analysis of climatic variability and characterization of groundwater quality including physico-chemical properties, hydrochemical composition and irrigation suitability assessment. The treatment performance of AgNPs/AC-NC nanocomposite filtration system is discussed in Sect. 6 through comparative analysis of groundwater quality parameters before and after treatment under different seasonal conditions. Statistical analysis is presented in Sect. 7. Finally, discussion and conclusions are presented in Sects. 8 and 9, respectively.

## Literature Review

Recent studies conducted between 2020 and 2024 have examined groundwater quality across different hydrogeological settings in Egypt, as summarized in Table 1^46–59^. These studies have mainly focused on hydrochemical characterization, drinking- and irrigation-water suitability, aquifer mapping, modelling, and health-risk assessment. The groundwater quality was evaluated using hydrochemical analysis coupled with water quality index (WQI), Piper and Gibbs plots in the study in^46^. Despite the fact that the study provided useful information on groundwater quality, it was limited to seasonal assessment only and no treatment or reuse pathways were recommended.

At a larger scale, groundwater resources in the Western Desert have been extensively documented in^47^, with a complete overview of its hydrogeology and hydrochemistry. The work provides a valuable understanding of groundwater resources and quality at a strategic level, however, it focuses on resource identification and regional supply, operates at a spatial scale, and, similar to^46^, lacks reuse interventions. In contrast, the work in^48^ investigated shallow groundwater quality at a local scale in El-Obour city, where a photocatalytic treatment method was used to improve water quality for reuse, making it the closest study to the present work in terms of including a treatment step. Nevertheless, the work was limited to a single-stage, one-time laboratory-scale process rather than a continuously operated field unit; thus, it lacked seasonal variations and posttreatment verification of water quality against irrigation suitability criteria.

With respect to aquifer exploration, the study in^49^ conducted in Siwa Oasis used electrical resistivity for aquifer potential mapping, whereas^50^ compiled geophysical survey examples in the southwestern desert and oases for aquifer mapping. Similarly, the work in^51^ for West Middle Upper Egypt used isotopic, geophysical, and hydrochemical methods to assess groundwater resources. Additionally^58^, combined vertical electrical soundings (VESs) with hydrochemistry to evaluate groundwater potential and suitability in the Minia region. While such geophysically driven studies are valuable for identifying aquifer potential, groundwater occurrence, and suitability status, they remain primarily exploratory and diagnostic.

Improvements in data analysis and modelling have also been applied to groundwater quality assessment. The work in^52^, conducted in El Kharga Oasis, combined hydrochemical and irrigation water indices (IWQIs) with machine learning and geographic information systems (GISs) to predict groundwater suitability for irrigation, whereas^57^ evaluated drinking-water risk at a regional level using the drinking water quality index (DWQI) and physicochemical and health criteria. However, this study contributes to the analytical perspective focused on prediction and classification using artificial neural networks (ANNs) rather than irrigation suitability assessment and reuse potential.

Several studies have applied hydrochemical and multivariate statistical techniques to evaluate groundwater quality. The work in^53^ in North Assiut and^56^ in West El Minia combined multivariate statistical analysis using principal component analysis (PCA) and hierarchical cluster analysis (HCA) with hydrochemical assessment to determine groundwater suitability for drinking and irrigation, whereas^55^ used major ions and trace elements to assess groundwater suitability in desert areas. Although these studies improved the interpretation of groundwater quality patterns and the statistical attribution of contamination sources, they remained primarily diagnostic and lacked posttreatment validation or reuse-oriented implementation. Similarly, the hyperarid assessment reported in^54^ provides valuable insight into water quality conditions in hyperarid environments. However, although it is framed as a simulation of surface and subsurface water quality, it is largely an index-based suitability evaluation rather than a process-based numerical model, with no direct intervention. This work, therefore, is beneficial for characterizing existing water conditions and hydrochemical behavior, without extending to treatment implementation or posttreatment validation.

In terms of the effects on human health risks^59^, analysed hydrochemical parameters and carcinogenic and noncarcinogenic health indices to assess drinking water safety in the El-Farafra Oasis. Although the study provided an important quantification of heavy metal exposure risks, it focused on risk characterization rather than risk reduction and did not include a treatment component to mitigate the identified contaminants.

In light of the above, although a plethora of research has focused on groundwater quality assessment, hydrochemical characterization, geophysical exploration, numerical modelling, and health risk evaluation throughout Egypt, integrated and application-oriented groundwater management studies remain limited. First, the majority of existing work has concentrated on groundwater diagnosis, classification of suitability for irrigation or drinking purposes, or mapping the potential of aquifers on a regional scale while overlooking mitigation through onsite intervention. In contrast, this study is positioned to fill this research gap by providing a single, operational framework integrating seasonal groundwater monitoring and quantitative suitability assessment using different water quality and irrigation indices rather than isolated single-purpose studies. Second, the work in this study is based on a campus-scale pilot system that integrates groundwater abstraction, treatment, and reuse, which are largely absent from the Egyptian groundwater literature. While there are some localized pilot treatment studies, they often lack performance evaluation under variable climatic conditions or posttreatment compliance with benchmark standards, and they focus mainly on single-stage laboratory-scale processes.

Third, and most importantly, the treatment approach proposed in this research utilizes an AgNPs/AC-NC medium that combines the high adsorption capacity and porous structure of activated carbon with the antimicrobial and catalytic properties of silver nanoparticles. Such a hybrid material is therefore expected to provide a dual treatment function, removing dissolved contaminants while simultaneously improving microbial reduction, thus addressing both chemical and biological water quality issues relevant to irrigation reuse. Finally, the proposed system incorporates an all-in-one solar PV system, transforming the treatment process into a sustainable and decentralized solution that connects groundwater reuse with renewable energy application under local climatic conditions.

**Table 1:** Literature review.

Ref	Study Context	Groundwater Assessment	Treatment	RES	Primary Application	Scale
^46^	Dayrout, Upper	WQI, Piper, Gibbs, hydrochemistry	×	×	Drinking & irrigation suitability	Local/urban
^47^	Kharga Oasis/Nubian Sandstone Aquifer	Hydrochemistry, GIS, WQI, irrigation indices, factor analysis	×	×	Drinking and irrigation suitability	Regional
^48^	El-Obour City, NE Cairo	Hydrochemical, microbial, heavy metals + photocatalytic treatment.	✓	×	Water reuse after treatment	Local/pilot
^49^	Siwa Oasis	Geophysical exploration, electrical resistivity, magnetic interpretation, hydraulic parameters	×	×	Aquifer potential mapping/land-use planning.	Regional
^50^	SW Desert & oases	Geoelectrical, hydrogeological, salinity assessment	×	×	Groundwater potentiality/aquifer delineation.	Regional
^51^	West Middle Upper Egypt	Isotopes, geoelectrical VES, hydrogeochemistry, irrigation indices	×	×	Groundwater recharge/source identification & irrigation suitability	Regional
^52^	El Kharga Oasis	Hydrochemistry, IWQI indices, machine learning, GIS	×	×	Irrigation suitability & groundwater management	Regional/oasis-scale
^53^	North Assiut	Hydrochemistry, irrigation indices, Piper/USSL/Wilcox/Gibbs, multivariate statistics	×	×	Drinking & irrigation suitability	Regional
^54^	Hyperarid	Physico-chemical analysis, SAR, SSP, Piper, chloride mass balance	×	×	Multiuse water quality: irrigation, livestock, industrial uses	Regional
^55^	Central Eastern Desert	Major ions & trace metals	×	×	Drinking, domestic, & irrigation suitability	Regional
^56^	West El Minia	Hydrochemistry, PCA, HCA, geostatistics, GIS mapping, irrigation indices	×	×	Drinking, domestic, & irrigation suitability	Regional
^57^	El Kharga Oasis	DWQI, health index, heavy metals, multivariate statistics, ANN, GIS	×	×	Drinking water quality & health risk assessment	Regional/oasis-scale
^58^	Minia region,	Geophysical VES, hydrochemistry, irrigation indices	×	×	Groundwater potential & drinking/irrigation suitability	Regional
^59^	El-Farafra Oasis/Nubian Sandstone Aquifer	Heavy metals, carcinogenic & noncarcinogenic health risk indices	×	×	Drinking water health risk assessment	Regional/oasis-scale
*	Abu Qir, Alexandria	Integrated seasonal physico-chemical, biological, & irrigation suitability assessment	✓	✓	Treated groundwater reuse for irrigation	Campus-scale pilot

✓ Indicates that it is covered in this reference and × indicates that it is not covered in this reference

^*^ Indicates that in the presented study

### Study area

**Fig. 1:**
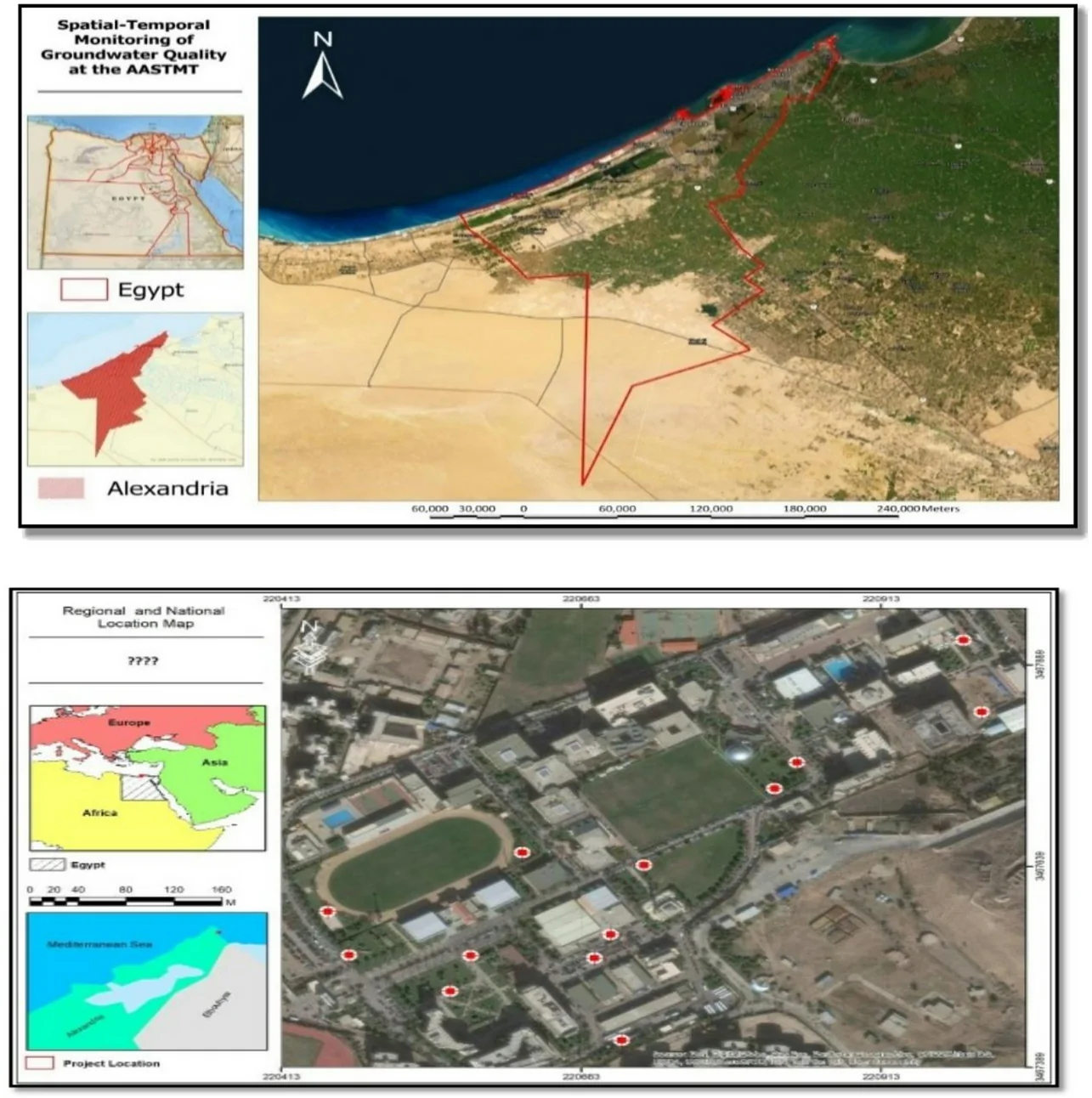
Spatial distribution of groundwater (**a**) temperature, (**b**) colour, (**c**) turbidity, and (**d**) pH. The maps were created by the authors using QGIS version 3.34 (QGIS Development Team, QGIS Geographic Information System, Open Source Geospatial Foundation Project, https://qgis.org). Satellite imagery was obtained from Google Earth (https://earth.google.com; imagery date: January 2021).

**Fig. 2:**
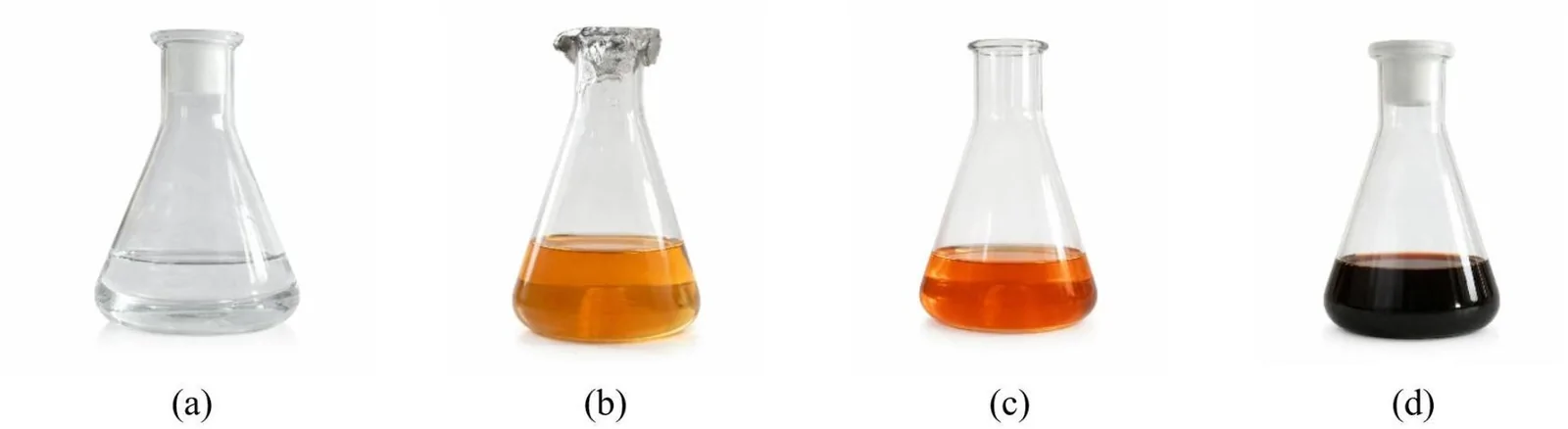
Preparation of silver nanoparticle (**a**) AgNO_3_ stock solution (**b**) olive leaf extract (**c**) AgNPs formation (**d**) AgNPs after 24 h.

This study was conducted in the Arab Academy for Science, Technology and Maritime Transport (AASTMT) campus is located in Abu Qir district, east of Alexandria city, and a full project report is available at^60^. The AASTMT covers approximately 25 acres, as depicted in Fig. 1, and the land is currently irrigated by 13 groundwater borehole , as shown in Tables 2 and Fig. 2^60^. The study area is situated in the Nile Delta coastal plain and is underlain by the Quaternary Nile Delta aquifer, one of the most important groundwater resources in northern Egypt^61^. The aquifer is mainly composed of unconsolidated sand and gravel deposits interbedded with clay lenses that were formed through the successive Nile sedimentation processes^61^. The borehole wells investigated are shallow groundwater wells with depths between 6 and 15 m below the ground surface as shown in Table 2, indicating that the samples collected were representative of groundwater from the shallow part of the local aquifer system. Due to the proximity of the study area to the Mediterranean Sea, the groundwater system may be affected by seawater intrusion processes, which can lead to increases in salinity indicators, such as the concentrations of sodium and chloride. Additionally, and due to proximity to industrial sites near the location, expected elevated values of metal hazards is also possible. The climate of Alexandria, Egypt, is characterized by hot summers and mild winters with moderate rainfall throughout the year. The temperature during the summer months can reach 35 °C to 40 °C, while winter temperatures are generally around 15 °C to 20°C^60^. Humidity levels are generally high because of the city’s coastal location on the Mediterranean Sea at latitudes and longitudes of 31° 12’ 20.7108’’ and 29° 55’ 28.2936’, respectively^60^.

**Table 2:** Locations and coordinates of the borehole wells^60^.

Latitude	Longitude	Well Number	Aquifer depth (m)	Location
31.30888	30.06532	1	15	Behind the Restaurant
31.30862	30.06518	2	12	In Front of the Power Station
31.3077	30.06545	3	6	In Front of the Garage
31.30822	30.06393	4	12	In Front of Hotel B
31.30862	30.0641	5	12	In Front of the Bank
31.3086	30.06303	6	12	In Front of the Entrance to the Academy
31.30908	30.06283	7	12	The Beginning of the Football Court
31.30978	30.06452	8	12	The End of the Football Court
31.31085	30.06688	9	12	Behind the Planetarium
31.31055	30.0667	10	12	In Front of the Clinic
31.31225	30.0683	11	12	In Front of the Marine Students Cafe
31.31145	30.06848	12	12	Next to the Faculty of Pharmacy
31.3075	30.0651	13	6	Inside the Arboretum

## Methodology

The present study aims to develop an integrated framework for groundwater monitoring, quality evaluation and treatment to improve its suitability for irrigation under climate-change scenario. The approach integrates field-based monitoring, laboratory analyses and applied treatment technologies under real operational conditions. The study focuses on capturing the spatial and temporal variations of groundwater quality, evaluating its suitability for irrigation using established indices and investigating a nano-based treatment solution for its improvement. The methodological framework comprises four main components: (i) groundwater sampling and analysis, (ii) solar powered groundwater abstraction, (iii) treatment unit design using advanced materials and (iv) performance evaluation based on water quality improvement and compliance with irrigation standards.

### Groundwater sampling and analysis

Physical, chemical and microbiological characterization of groundwater samples is essential for determining the spatial and temporal variations in quality. The physical parameters included temperature, colour, turbidity, electrical conductivity, and total dissolved solids^60^. Chemical analyses covered major cations and anions, nutrients, trace elements, and chemical oxygen demand, whereas microbiological indicators were used to assess potential health and irrigation risks. Characterization of water samples is essential because it will help in (1) determining the quantitative and qualitative distributions of pollutants in the study area, (2) defining the effects of climate change (temperature and precipitation) on groundwater quality, (3) determining the suitability of groundwater for irrigation purposes through calculations of water indices, and (4) proposing the best treatment to enhance water quality.

A total of 156 groundwater samples were collected throughout the study period (13 wells × 4 seasons × 3 field replicates). The same borehole wells were sampled during four seasonal campaigns conducted in winter (15–17 January 2022), spring (10–12 April 2022), summer (5–7 July 2022), and autumn (20–22 October 2022). For each well and sampling campaign, three independent groundwater samples were collected to improve data reliability and account for potential sampling variability. The investigated wells are shallow borehole wells with depths ranging from 6 to 15 m below the ground surface, as presented in Table 2. Prior to sample collection, each well was purged by pumping until the field parameters, including the pH, EC, and temperature, stabilized to ensure that the collected samples were representative of aquifer conditions rather than stagnant water within the well casing. Each well was operated under normal irrigation conditions for approximately 5 min before sampling. Following purging, groundwater samples were collected directly from the pump discharge line into precleaned sampling containers.

For microbiological and physicochemical analyses, all samples were collected in presterilized 250 mL polypropylene bottles and acid-washed 1 L high-density polyethylene (HDPE) bottles, respectively. Samples designated for trace metal analyses (Cu, Fe, Ca, Mg, Na, and K) were preserved by acidification with ultrapure HNO₃ to a pH < 2 and stored at approximately 4 °C until analysis. Samples for anion determination (Cl^-^, SO₄²^-^, NO₃^-^, NO₂^-^, and F^-^) were collected in HDPE containers and refrigerated at 4 °C without acidification. The microbiological samples were analysed within 6 h of collection and transported to designated laboratories under cooled conditions. COD samples were preserved according to standard analytical procedures, while other field parameters including pH, EC, DO and temperature were measured immediately after sampling using calibrated portable measuring instruments.

All the samples were labelled using unique, systematic identifiers specifying the well number, season, replicate number and sampling date. A chain-of-custody procedure was maintained throughout the sampling and analytical process whereby sample collection, preservation, transport, receipt and storage were documented using standardized records. The samples were transported to the laboratory immediately after collection in insulated coolers containing ice packs to maintain a temperature of approximately 4 °C. Upon arrival at the laboratory, all samples were logged, assigned a laboratory identification number and stored under the appropriate preservation conditions until analysis. All analyses in this study were carried out in accordance with the Standard Methods for the Examination of Water and Wastewater^62^.

### Green preparation of AgNPs

This stage dealt with the preparation of AgNPs using a plant-based reducing and stabilizing agent, following a four-stage process. First, the olive leaves were separated and rinsed thoroughly with distilled water and dried at 25 °C. The dried leaves were ground, and 7.5 g was dissolved in 100 ml of demineralized water, where the mixture was left to boil with continuous stirring for 15 min, after which it was filtered through filter paper. The filtrate was diluted to 250 ml with demineralized water to produce an olive leaf extract (OLE). Second, the silver nitrate stock is prepared by weighing 0.34 g of silver nitrate (0.02 M) in 100 ml of demineralized water, resulting in a clear AgNO3 solution, as depicted in Fig. 2(a). In the third stage, silver nanoparticles (AgNPs) were synthesized by adding 20 ml of AgNO_3_ to 100 ml of OLE with continuous stirring for 20 min. The olive leaf extract turned yellowish to light brown, as illustrated in Fig. 2(b). Upon mixing, the color gradually changed to a reddish-brown suspension, indicating the formation of AgNPs, as demonstrated in Fig. 2(c). The solution turned to deep brown color after standing for 24 hr at room temperature indicating the formation of AgNP as shown in Fig. 2(d). Finally, in the fourth stage, 50 g of activated carbon was added to 100 mL of olive leaf extract to prepare a silver nanocomposite (AgNCs) coated on activated carbon. The mixture was stirred vigorously for 2 hr, then the solution was sonicated at 100 Hz for 40 min and finally dried in an oven at 70 °C.

### Characterization of the nanocomposite materials (NCMs)

**Fig. 3:**
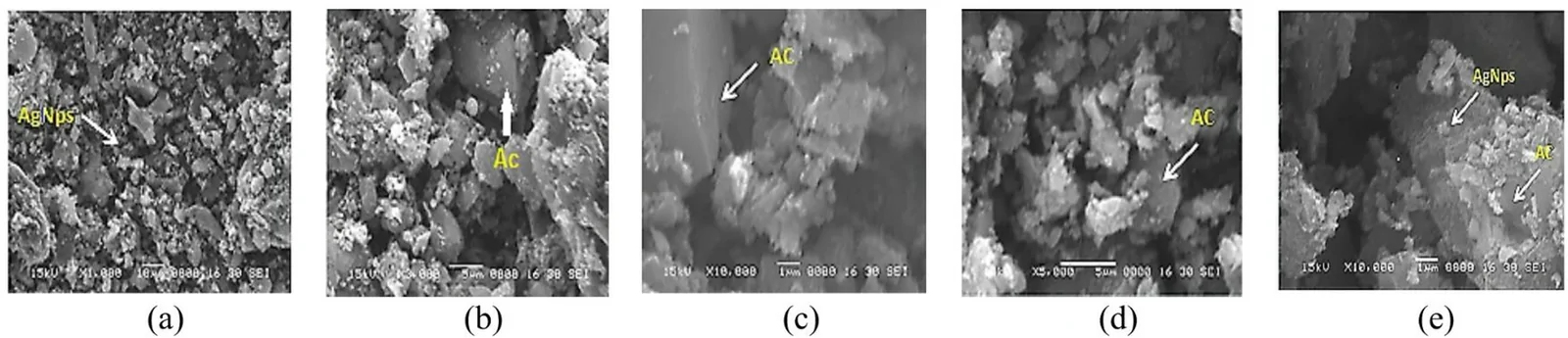
SEM Micrographs of AgNPs and AgNPs/AC-NC with various scales (**a**) 1000 x, (**b**) 3000 x, (**c**) 10,000 x, (**d**) 5000 x and (**e**) 10,000 x.

**Fig. 4:**
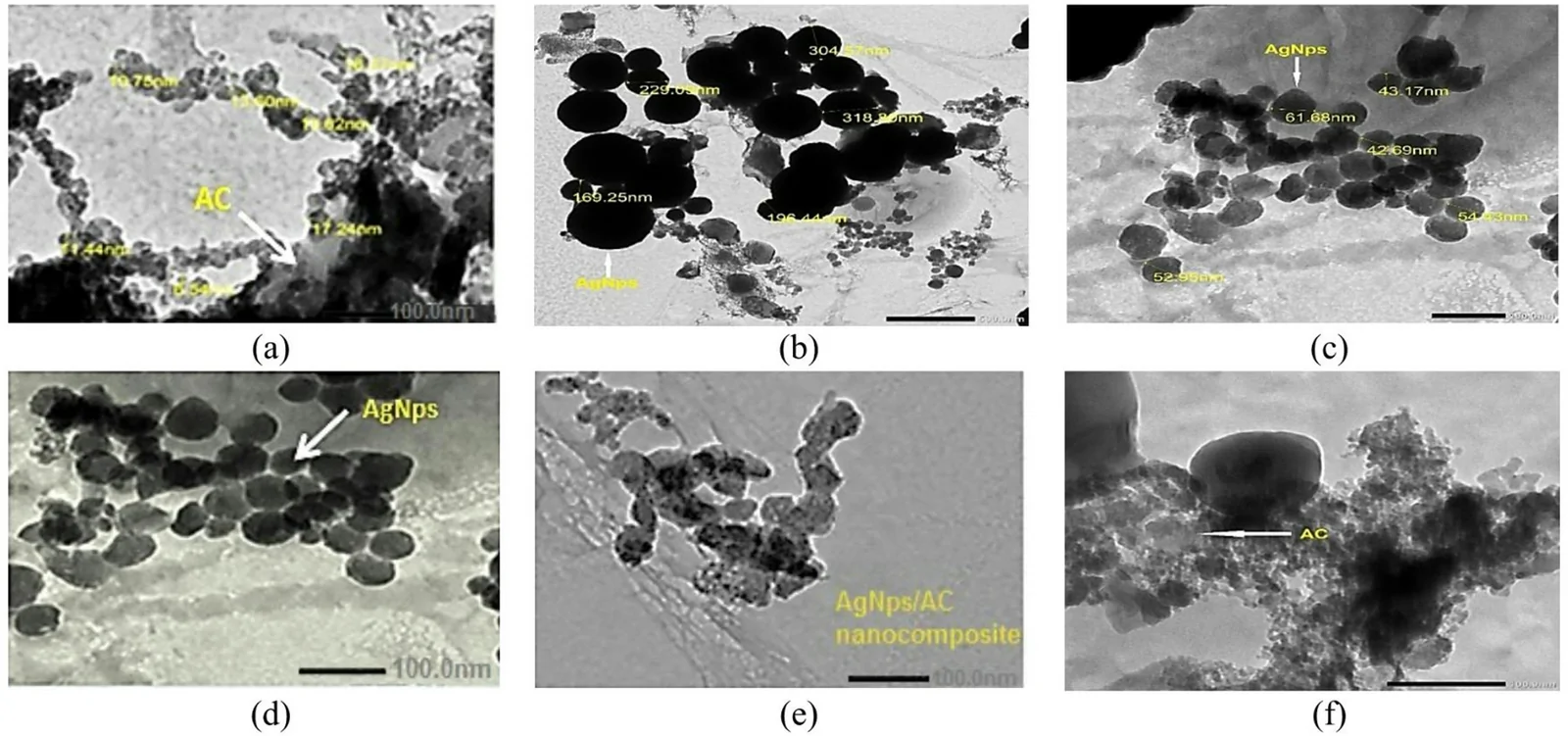
TEM micrographs of synthesized AgNPs and AgNPs/AC-NC at different scales (**a**) 100 nm, (**b**) 500 nm, (**c**) 100 nm, (**d**) 100 nm, (**e**) 100 nm and (**f**) 100 nm.

**Fig. 5:**
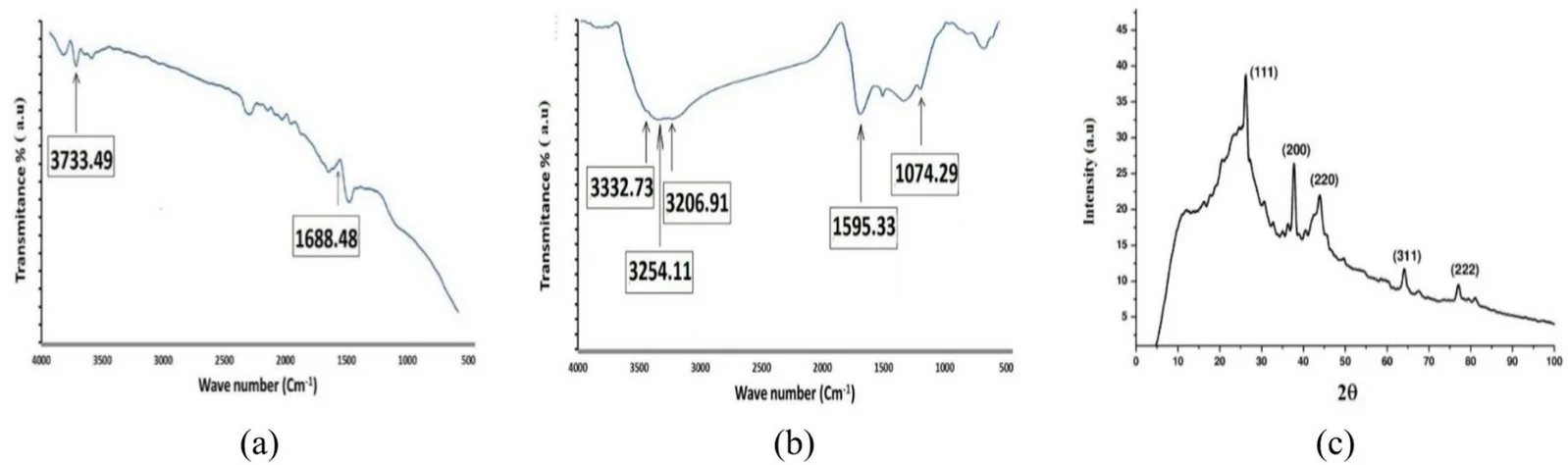
FTIR Spectra of (**a**) AgNPs and (**b**) AgNPs/AC-NC, and (**c**) XRD pattern of AgNPs/AC-NC.

Various characterization techniques were employed to successfully confirm the synthesized NCMs and evaluate the structural, chemical and morphological properties of the prepared AgNCs in terms of their distribution on the activated carbon surface and the presence of active functional groups for adsorption and antimicrobial activity. These include scanning electron microscopy (SEM), transmission electron microscopy (TEM), Fourier transform infrared (FTIR) spectroscopy, X-ray diffraction (XRD), and energy dispersive X-ray (EDX) analysis^60^. The morphology and elemental composition of the nanocomposites were analysed using SEM equipped with an energy dispersive X-ray (EDX) system. The particle size, shape, and dispersion of the synthesized AgNPs were determined using TEM. The crystal structure and phase composition of the nanocomposites were analysed using an X-ray diffraction (XRD) instrument equipped with an advanced diffractometer with a Cu target. The instrument was operated with CuKα radiation (λ = 1.54 A) generated at 40 kV and 40 mA^60^. Moreover, the surface functional groups and chemical interactions present in the nanocomposites were investigated using FTIR to identify the organic functional groups present, as well as to investigate the molecular interactions between the adsorbed molecules and the nanoparticles. The representative SEM, TEM, and FTIR results obtained for the synthesized nanocomposite materials are shown in Fig. (3, 4 and 5).

The activated carbon exhibited a rough and porous surface morphology with irregular cavities. The bright particles on the carbon surface indicated the presence and deposition of silver nanoparticles. The SEM micrographs confirmed the successful distribution of the AgNPs on the activated carbon matrix, while preserving the porous structure of the support material. The formation of AgNPs on the activated carbon surface was further confirmed by TEM analysis. The synthesized silver nanoparticles were spherical to almost spherical in shape, with a mean diameter of 22 ± 6 nm and particle sizes between 15 and 35 nm, based on the measurement of 100 particles. Some localized aggregation was observed, but most of the particles still attached to the carbon matrix, confirming the successful formation of the AgNPs/AC-NC.

Besides, XRD analysis showed characteristic diffraction peaks at 2θ = 38.1°, 44.3°, 64.5°, and 77.4°, which correspond to the (111), (200), (220), and (311) crystal planes of face-centered cubic silver, respectively. In addition, the broad diffraction feature in the pattern is attributed to the amorphous structure of the activated carbon support. Additionally, the FTIR spectrum of activated carbon showed absorption bands at 3733.49 and 1688.48 cm^− 1^, corresponding to O–H and C = O functional groups, respectively. The introduction of silver nanoparticles led to the appearance of characteristic bands at 3332.73, 3254.11, 3206.91, 1595.33 and 1074.29 cm − 1. These bands were attributed to O–H stretching vibrations, aromatic C = C vibrations and C–O stretching groups, confirming the successful synthesis of the AgNPs/AC-NC.

### Modified AgNPs/AC-NC filtration system

**Fig. 6:**
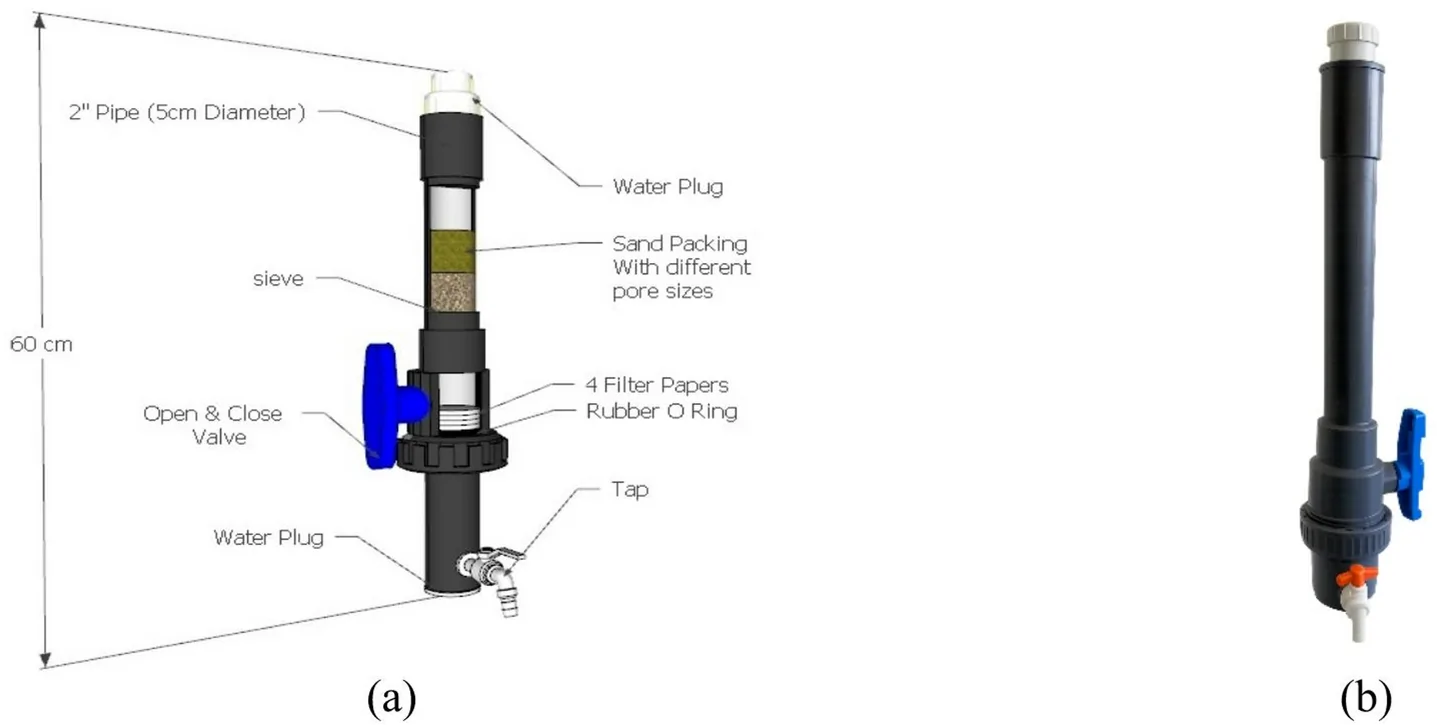
Proposed Ag/AC NC - Amended Filter (**a**) designed column, (**b**) actual columns.

Groundwater was introduced into the designed column shown in Fig. 6(a–b) through the upper opening and allowed to pass through the AgNP/AC nanocomposite filtration medium before being collected through the bottom outlet for subsequent analysis. The flow rate was initially set to approximately 3 L/min; however, preliminary hydraulic testing showed that this produced an empty-bed contact time (EBCT) of only ~ 5.9 s and inadequate treatment performance. Thus, the operating flow rate was reduced to 0.05 L/min (50 mL/min), measured three times per sample and maintained at this value throughout the experiments, to ensure sufficient contact time between groundwater and the AgNP/AC medium. For a packed bed height of 15 cm and a bed volume of 294 cm^3^, the corresponding EBCT was 5.88 min, while the superficial velocity and hydraulic loading rate were 2.55 cm/min and 1.53 m/h, respectively.

The investigation examined the performance of the AgNPs/AC nanocomposite at different dosage levels ranging from 0.2 to 1.0 g/L. In each treatment, 1000 mL of groundwater was passed through the column at room temperature using the specified AgNP/AC nanocomposite dosage, and the filtration experiments were performed as single-pass treatment tests. Preliminary dosage optimization experiments indicated that increasing the AgNP/AC dosage improved treatment performance by increasing the availability of active adsorption sites. The optimum dosage was 1.0 g/L, which gave the best overall improvement in groundwater quality parameters while maintaining reasonable material utilization; this dosage was therefore adopted for all subsequent treatment experiments reported in this study. Notably, the primary objective of the filtration experiments was to evaluate the treatment performance of the AgNP/AC nanocomposite at different dosage levels, and the column dimensions were accordingly fixed at 5 cm in diameter and 15 cm in height throughout. Long-term operational parameters, including breakthrough behaviour, adsorption-capacity exhaustion, regeneration potential, reusability, Ag leaching, and filter lifetime, were not investigated in the present study and are recommended for future work focusing on practical scale-up and long-term field operation.

### Solar-powered pumping system

**Fig. 7:**
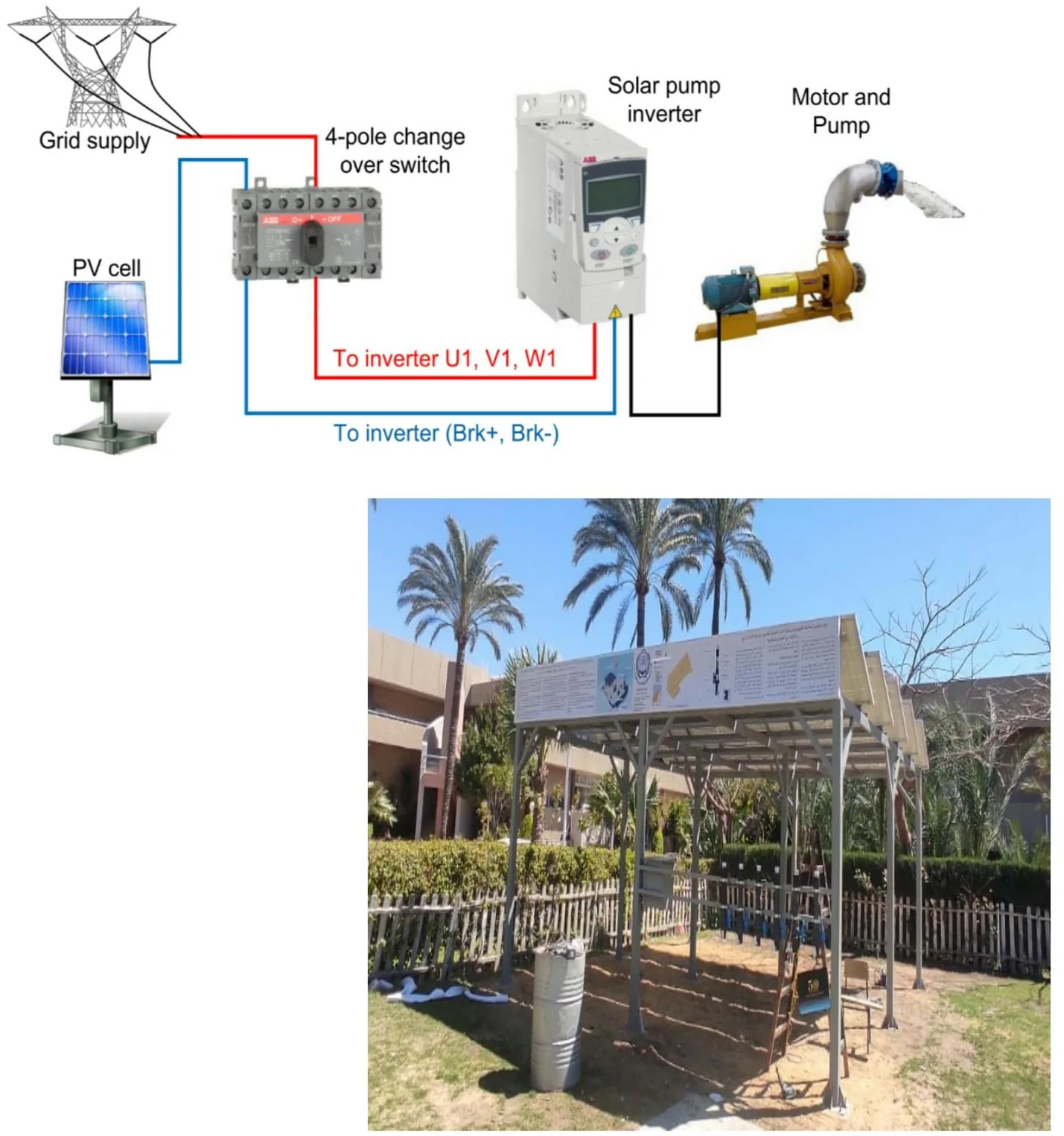
Electrical pumping system (**a**) block diagram and (**b**) actual system.

The solar PV system serves as the sustainable energy source for the three-phase pumping system, whose main function is to lift water from the bore well and deliver it to the AgNP/AC nanocomposite filtration column for treatment^63,64^. In this case, the solar PV panels convert sunlight into electrical energy, which is then transformed by the solar inverter for pump operation. As depicted in the block diagram of Fig. 7, the main components are the PV panels, an inverter module, and a four-pole change-over switch when the pump is operated directly from the electrical AC grid^65^. Table 3 shows the specifications and ratings of the implemented PV system; accordingly, a total of 15 series-connected panels are installed such that the system generates a maximum power output of 6,824 watts under ideal solar conditions at 600 V^60^.

**Table 3:** PV system specifications and ratings.

Element	Parameter	Value/specs
PV modules	PV panel	JA Solar Mono − 455 W
	Efficiency	21%
	Open-Circuit Voltage $\:{V}_{oc}$	53.87 V
	Max. Power Point Voltage$\:{V}_{MPP}$	45.83 V
	Short Circuit Current $\:{I}_{Sc}$	10.56 A
	Max. Power Point Current $\:{I}_{MPP}$	9.93 A
	Dimensions (mm)	2180 $\:\times\:$996 $\:\times\:\:$40
Pump	Output power	3hp
	Supply current	6 A
	Pump speed	2830 rpm
	Power factor	0.71
	Ingress Protection	IP68
	Thrust load	3000 N
	Minimum pump flow	0.3 m/s
Inverter	ACS355	0.3–18.5 kW
	Frequency	50/60 Hz
	Output Current	5.4 A

### Performance evaluation metrics

The effectiveness of the integrated monitoring and treatment system was evaluated using several parameters. The removal efficiency of important physical, chemical, and microbiological parameters was calculated as the difference between influent and effluent concentrations. The suitability of groundwater for irrigation was also evaluated using water quality indices calculated before and after treatment. The quality of treated water was also compared with national and international standards for irrigation water to assess compliance and suitability for reuse. Operational stability was also evaluated by comparing the consistency of treatment performance across seasons and operating conditions.

With respect to water quality indices, salinity, sodicity, alkalinity and toxicity criteria were used to determine the quality of the water for irrigation. Additionally, the NA%, SAR, RSC, expected PI of soil, PS, KR, chloroalkaline indices (CAI1 and CAI2), magnesium hazard and TH were calculated using Eq. (1) to (8)^66–73^.

1 $$\:SAR=\frac{{Na}^{+}}{\sqrt{\frac{{Ca}^{2+}+{Mg}^{2+}}{2}}}$$

2 $$\:RSC=({\mathrm{H}\mathrm{C}\mathrm{O}}_{3}^{-}\:+\:{\mathrm{C}\mathrm{O}}_{2}^{-3})\:-\:({Ca}^{+2}\:+\:{Mg}^{2+})$$

3 $$\:Na\%=\left(\frac{{Na}^{+}+{K}^{+}}{{Na}^{+}+{K}^{+}+{Mg}^{2+}{Ca}^{+2}}\right)\times\:100$$

4 $$\:PI=\frac{{Na}^{+}+\sqrt{{HCO}_{3}^{-}}}{{Mg}^{+2}+{Ca}^{+2}+{Na}^{+}}$$

5 $$\:PS={Cl}^{-}+0.5\times\:{SO}_{4}^{-2}$$

6 $$\:CAI1=\frac{{Cl}^{-}-\left({Na}^{+}+{K}^{+}\right)}{{Cl}^{-}}$$

7 $$\:CAI2=\frac{{Cl}^{-}-\left({Na}^{+}+{K}^{+}\right)}{\left({SO}_{4}^{-2}+{\mathrm{C}\mathrm{O}}_{2}^{-3}+{\mathrm{H}\mathrm{C}\mathrm{O}}_{3}^{-}+{\mathrm{N}\mathrm{O}}_{3}^{-}\right)}$$

8 $$\:TH=2.5{Ca}^{+2}+4.1{Mg}^{+2}$$

## Results & analysis

### Climatic variability

Climatic conditions in Alexandria during the study period were analysed to examine their potential influence on seasonal groundwater quality variations. The analysis included air temperature and precipitation patterns, as depicted in the following subsections.

### Air temperature and precipitation

**Fig. 8:**
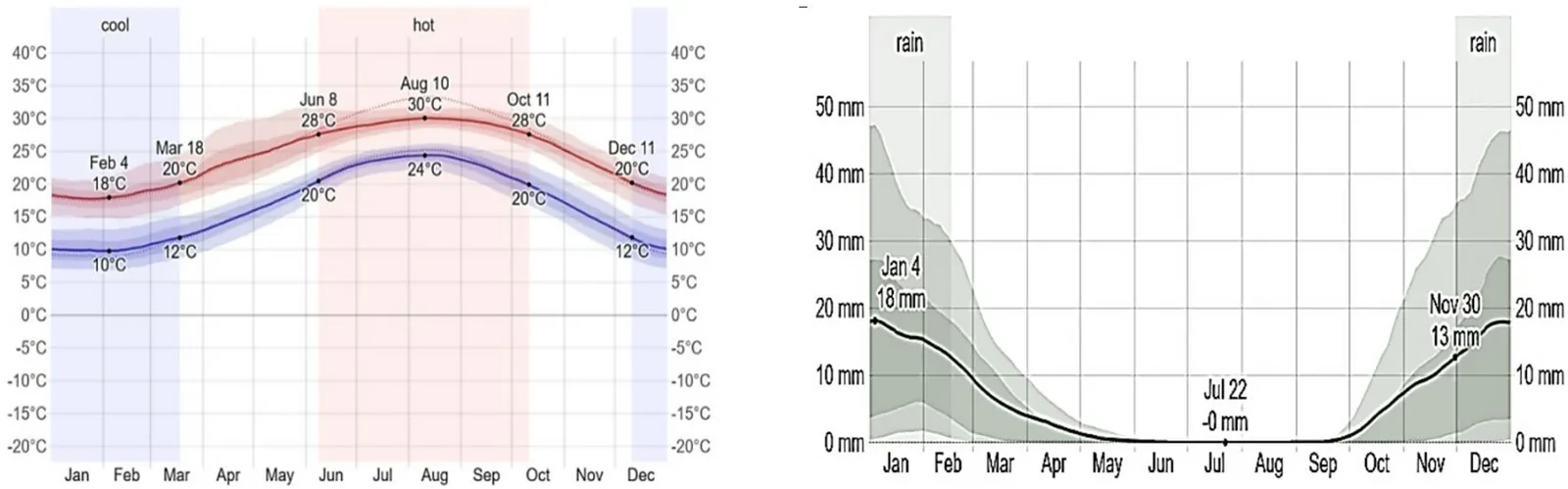
Daily average (**a**) Perceived temperatures (WeatherSpark.com), (**b**) Average rainfall (Solid Line).

The daily temperature variation in Fig. 8(a) clearly shows a seasonal variation, which is characteristic of Mediterranean coastal areas. The hot season lasted from early June to mid-October, during which the average daily maximum temperature was above 28 °C, with a maximum of approximately 30 °C in August. On the other hand, the cold season lasted from mid-December to mid-March, during which the average daily maximum temperature was less than 20 °C, with the lowest temperatures occurring in January (10–18 °C). The precipitation was strongly seasonal, with rainfall occurring between November and February, as indicated in Fig. 8(b), which represents the average rainfall accumulated over the course of the sliding 31-day period centered on the day in question, with 25th to 75th and 10th to 90th percentile bands. The rainfall was highest in January and March, while there was little to no rainfall between April and September, as shown in Table 4. The total annual rainfall in 2022 was approximately 282 mm. The fact that rainfall is most concentrated during the winter season indicates that recharge and dilution processes are also most likely to occur during this season, while the salinity levels are high during the summer season because of high evaporation rates and low recharge.

**Table 4:** Monthly total precipitation over Alexandria during 2022^60^.

Month	Mean	Min	Max	Sum
	(mm)			
Jan	4.1545	0	24.89	128.79
Feb	1.0614	0	9.91	29.72
Mar	2.3271	0	18.03	72.14
Apr	0	0	0	0
May	0	0	0	0
Jun	0	0	0	0
Jul	0	0	0	0
Aug	0	0	0	0
Sep	0	0	0	0
Oct	0.3723	0	4.06	11.17
Nov	0.2963	0	3.05	8.89
Dec	0.9997	0	19.81	30.99
Annual Mean	0.7739	0	24.89	281.7

### Groundwater quality characterization

#### Physicochemical Characteristics

**Fig. 9:**
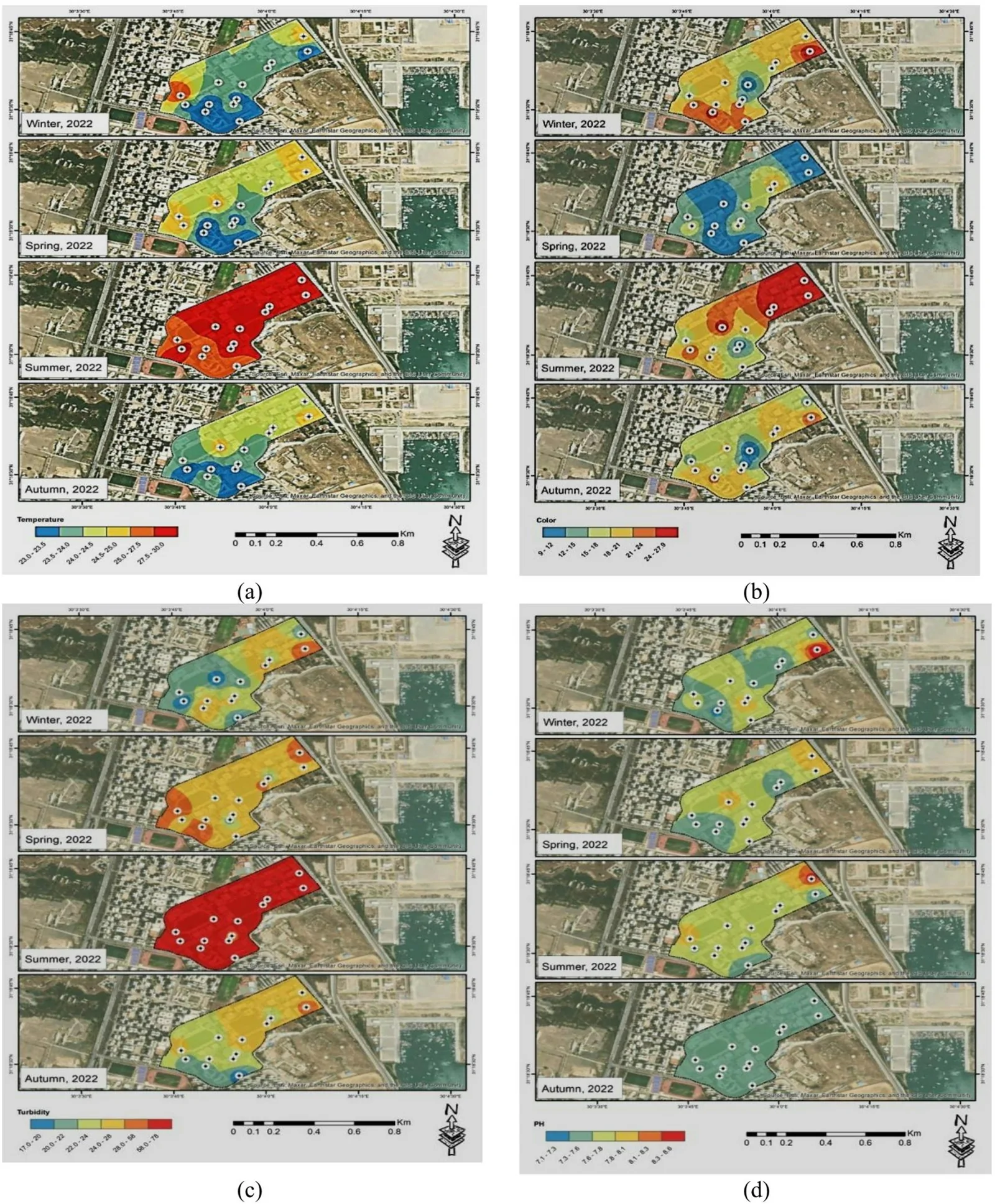
Spatial distribution of (**a**) Temperature, (**b**) Colour, (**c**) Turbidity, and (d) pH of the groundwater samples. The maps were created by the authors using QGIS version 3.34 (QGIS Development Team, QGIS Geographic Information System, Open Source Geospatial Foundation Project, https://qgis.org). Satellite imagery was obtained from Google Earth (https://earth.google.com; imagery date: January, April, July, and October 2021 for winter, spring, summer and autumn respectively).

The recorded temperatures of the groundwater samples during the study period were within the normal range. However, during the summer season, it was higher and varied between 26.5 and 29 °C, as shown in Fig. 9(a)^60^.

In terms of color, the color values differed between 3 and 28 Pt-Co units across seasons, as indicated in Fig. (9(b)), ranging from 7 to 28 Pt-Co units in winter, 3–22 Pt-Co units in spring, 9–28 Pt–Co units in summer, and 6 to 25 Pt–Co units in autumn. While most samples complied with the permissible limit (≤ 15 Pt–Co units), several wells exceeded this threshold during winter, summer, and autumn. Spring exhibited relatively low color values, with values falling below the permissible limit of^41^. Elevated color levels may indicate the presence of dissolved organic or particulate matter. On the other hand, the turbidity values exceeded the permissible limit of 5 NTU in all samples, with the highest values recorded in summer as per Fig. 9(c). Increased turbidity during warmer months may reflect enhanced particulate suspension and microbial activity. In contrast, the average TDS concentrations were 392.3 mg/L in the winter, 608.1 mg/L in the spring, 434.1 mg/L in the summer, and 371.2 mg/L in the autumn. Spring had the highest average TDS values, whereas autumn had the lowest. During winter and autumn, the majority of wells fell within the optimal salinity range of less than 450 mg/L. In contrast, all studied wells were classified as moderate in spring, versus 61.5% in summer, ranging from 450 to 2000 mg/L. While higher summer evaporation may increase dissolved solids, the recorded spring maximum suggests that TDS was not solely controlled by evaporation but may also be influenced by leaching of accumulated salts, agricultural return flow, fertilizers, and possible groundwater–seawater interactions in the coastal Abu Qir area. Despite higher evaporation, the lower summer average may reflect localized dilution from irrigation return water or changes in abstraction and recharge conditions. Yet, all recorded TDS values remained within acceptable irrigation limits across all seasons. With respect to pH, the quality of groundwater was evaluated through a comparison to the standard guidelines for pH set by^42^, which recommends pH level between 6.5 and 8.4. The studied groundwater reveals pH levels ranging from 7.1 to 8.6, indicating slightly basic conditions that are suitable for irrigation, as shown in Fig. 9(d).

#### Major ions and hydrochemical composition

**Fig. 10:**
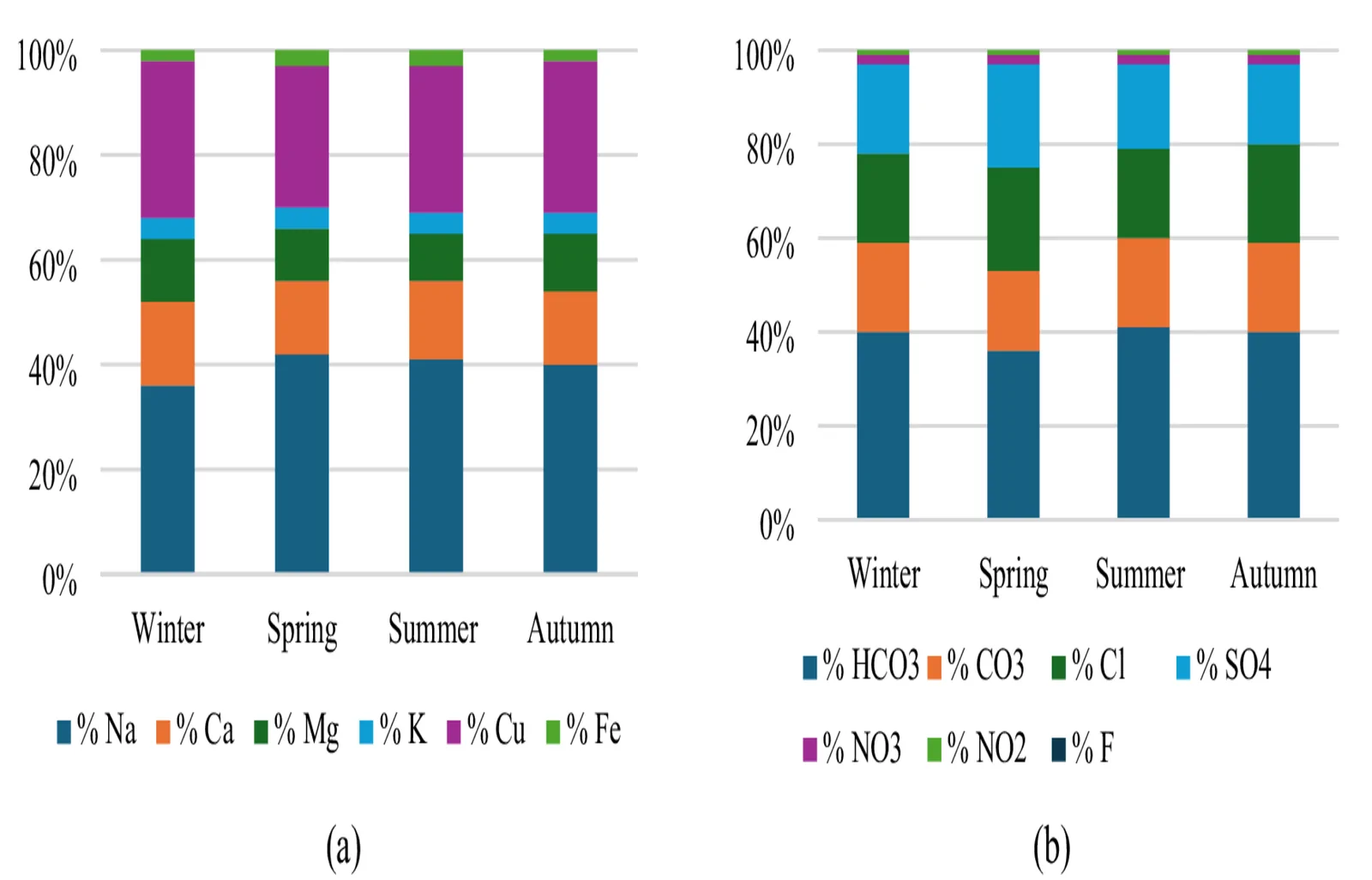
Major ion concentrations (**a**) cations; (**b**) anions.

The seasonal percentage dominance of major cations and anions is illustrated in Fig. 10(a–b). Bicarbonate contributed between 36 and 41% to the total dominance of anions during the season, whereas nitrite (NO₂^-^) and fluoride (F^-^) contributed the least with values less than 1%, where the corresponding seasonal distributions of major anions presented in Fig. 10(b). The dominance pattern of the major cations shown in Fig. 10(a), on the other hand, was Na⁺, followed by Ca²⁺, Mg²⁺, and K⁺, whereas that of anions was HCO₃^-^, then CO₃²^-^, Cl^-^, SO₄²^-^, NO₃^-^, NO₂^-^, and F^-^, suggesting that a sodium-bicarbonate water type prevails in the region, with a possible influence from seawater intrusion given the coastal location of the study area.

Regarding pH, values ranged from 7.1 to 8.6, indicating a slightly alkaline nature; most samples fell within the recommended irrigation range (6.5–8.4), with a few slightly exceeding the upper limit. The concentration of calcium varied from 15.3 to 28 mg/L, and magnesium between 7.77 and 21.04 mg/L across different seasons. Both Ca²⁺ and Mg²⁺ concentrations remained within the permissible irrigation limits; however, the magnesium hazard expressed by the MH index exceeded the recommended threshold in several wells. On the other hand, compared with those in winter (52.42 mg/L) and autumn (56.52 mg/L), the sodium concentrations in spring (68.27 mg/L) and summer (63.45 mg/L) increased seasonally. Despite this increase, the sodium values remained within acceptable irrigation standards.

Potassium concentrations ranged from 4.36 to 8.39 mg/L seasonally and met irrigation standards in all wells. The chloride concentrations varied from 59.08 to 98.2 mg/L during the entire period of investigation and remained below the maximum permissible standards. The fluoride concentrations varied from 0.71 to 0.96 mg/L and satisfied national and international standards. NO₃^-^ showed the least seasonal variation and remained below the FAO irrigation guideline of ~ 50 mg/L in all wells throughout the investigation period. Wells 10–13 had relatively high nitrate concentrations of up to ~ 15 mg/L which may indicate localized agricultural influence and should be monitored, however the values were within the suitable range for irrigation. Nitrite values showed considerable variation seasonally, especially in summer, possibly because of agricultural or domestic wastewater contamination. Bicarbonate concentrations varied from 143.53 to 197.8 mg/L, with slightly higher values in summer (mean 180.9 mg/L). Carbonate values exceeded the irrigation guideline standards in most wells, possibly increasing alkalinity and affecting sodium adsorption ratios. The sulphate concentrations varied from 56.7 to 98.6 mg/L and remained well below the permissible standards.

## Treatment performance evaluation

### Physical water quality improvement

**Fig. 11:**
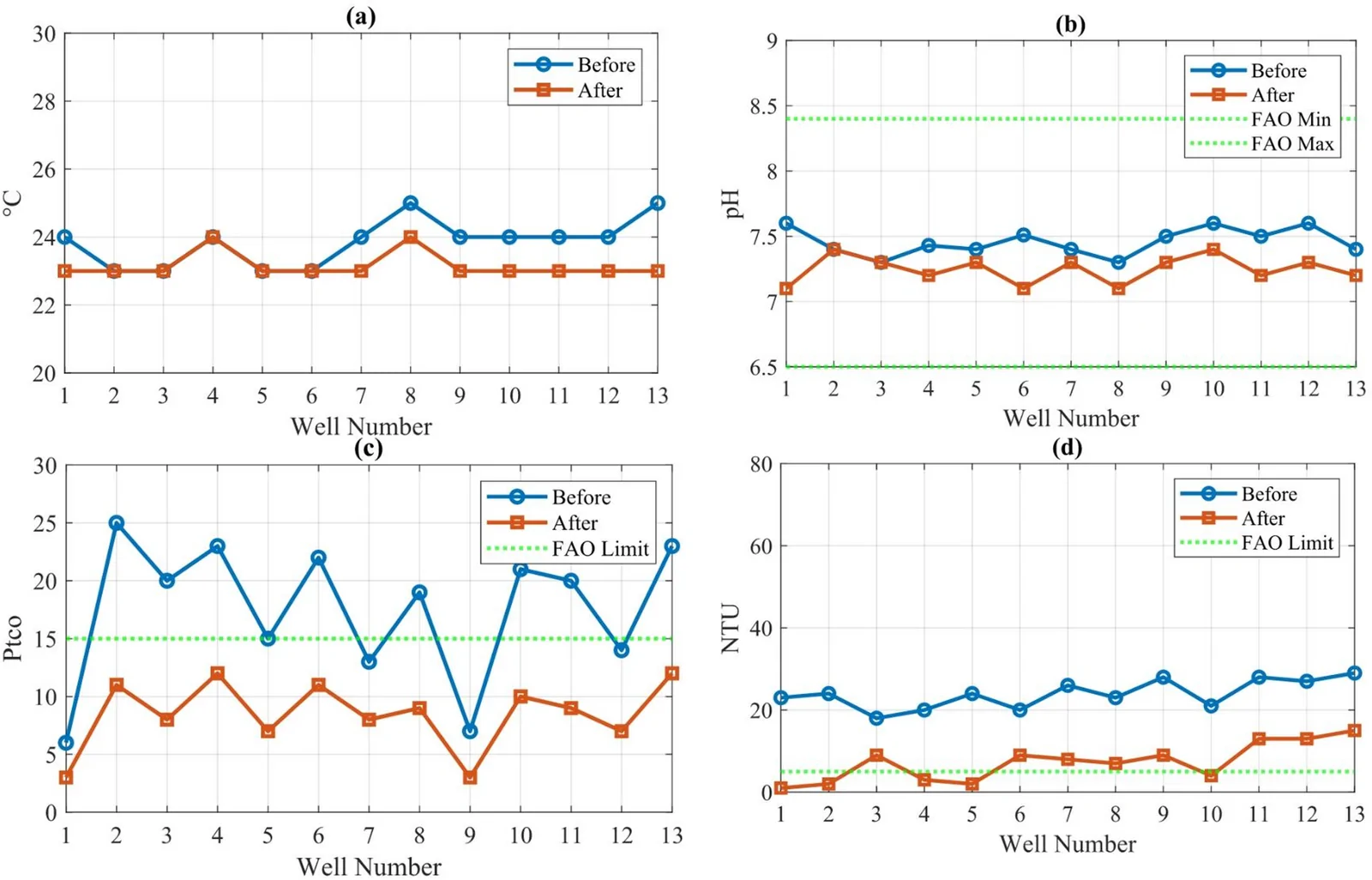
Physical water quality parameters before and after treatment for Autumn (**a**) Temperature, (**b**) pH, (**c**) Colour, and (**d**) Turbidity.

**Fig. 12:**
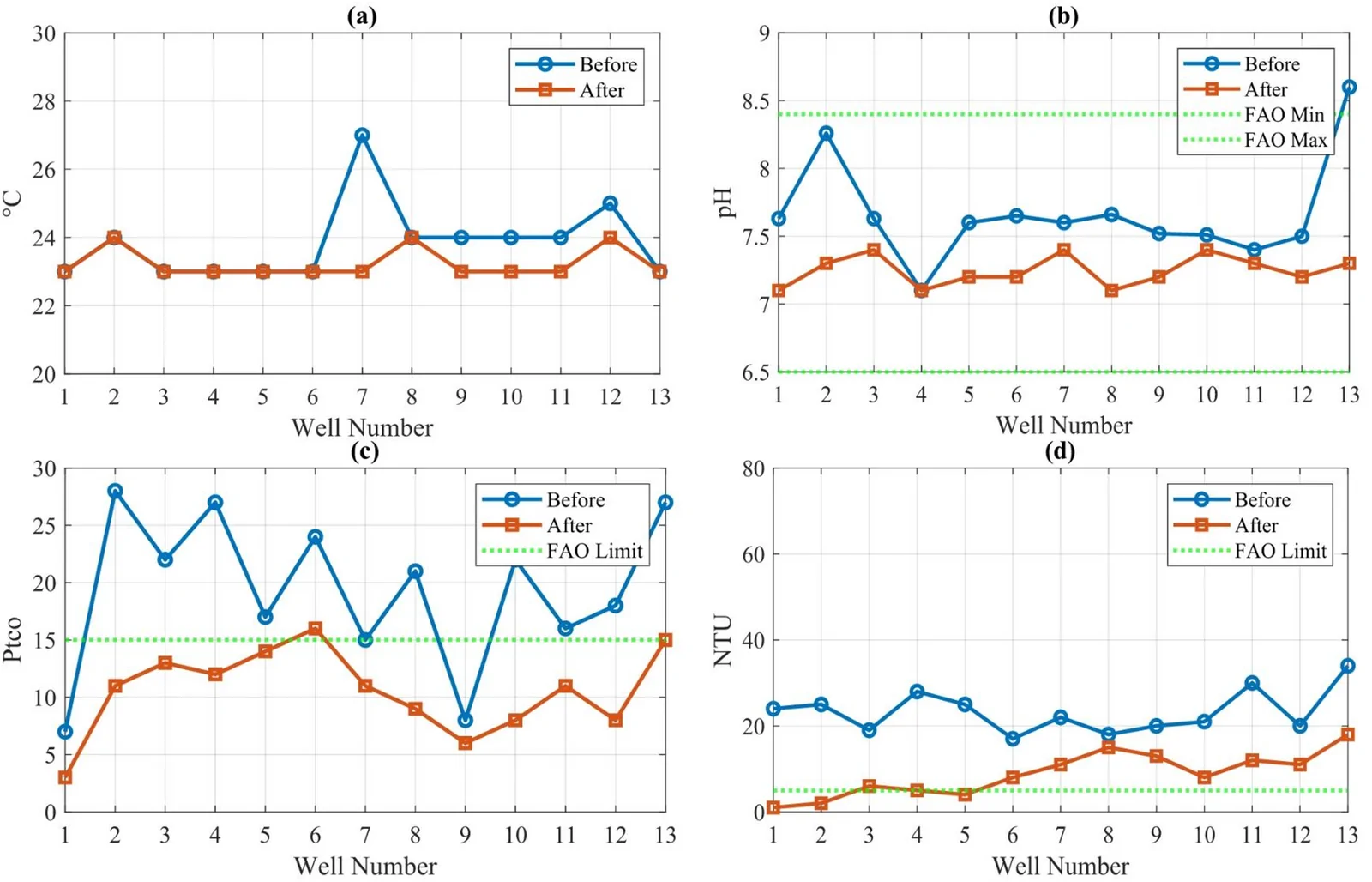
Physical water quality parameters before and after treatment for winter (**a**) Temperature, (**b**) pH, (**c**) Colour, and (**d**) Turbidity.

**Fig. 13:**
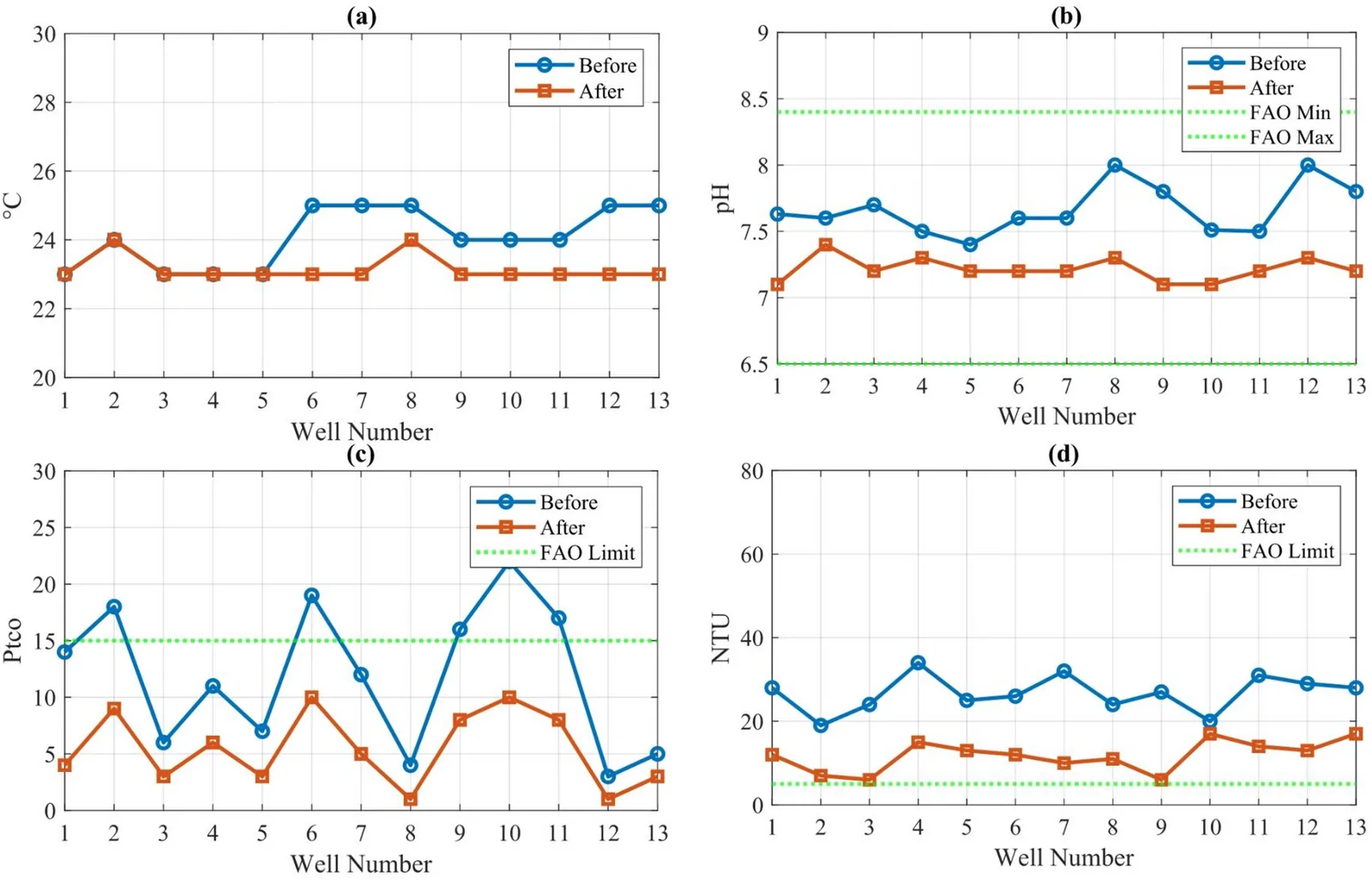
Physical water quality parameters before and after treatment for spring (**a**) Temperature, (**b**) pH, (**c**) Colour, and (**d**) Turbidity.

**Fig. 14:**
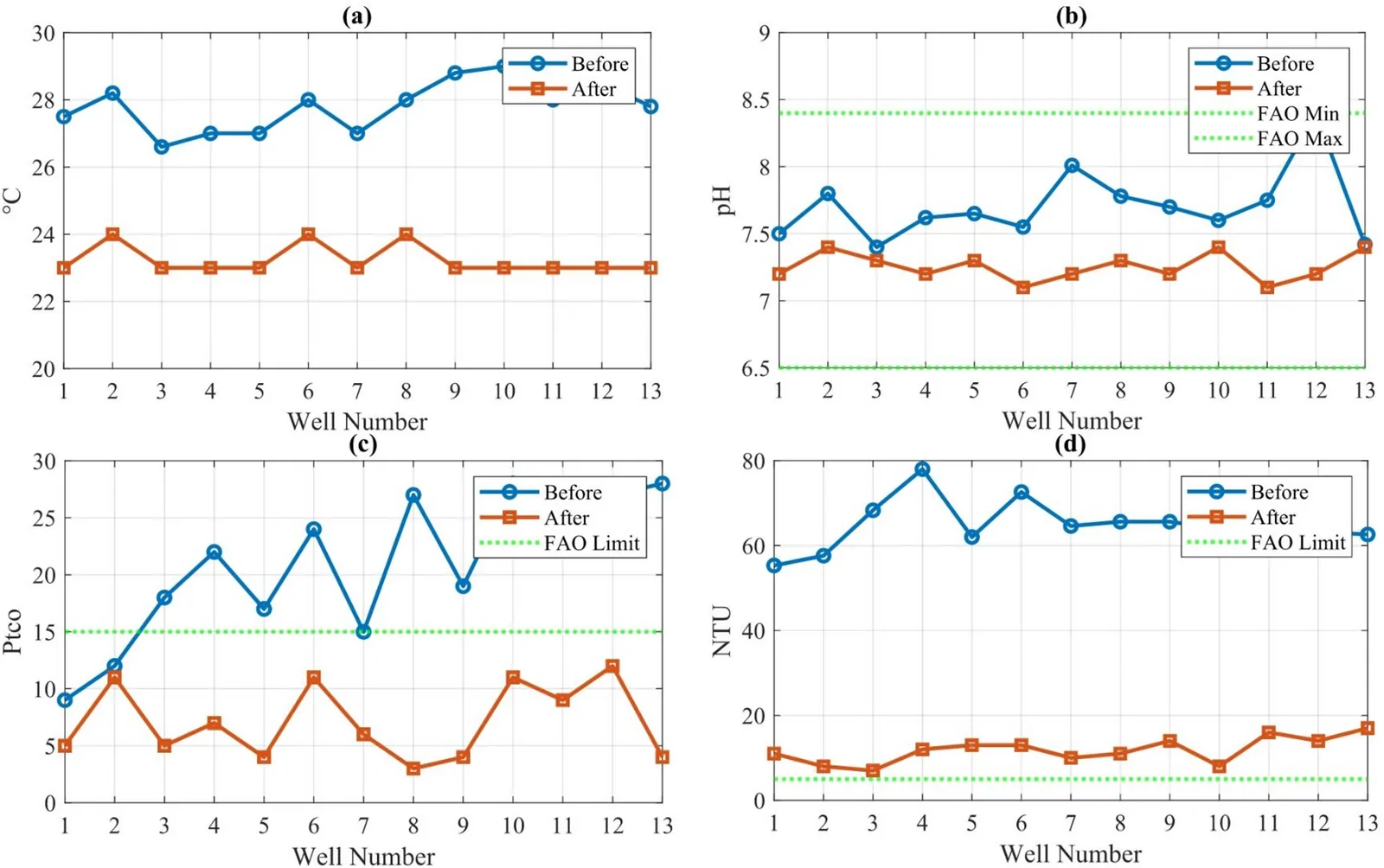
Physical water quality parameters before and after treatment for summer (**a**) Temperature, (**b**) pH, (**c**) Colour, and (**d**) Turbidity.

The AgNP/AC nanocomposite filtration system improved the physical groundwater quality parameters in all wells and seasons, as shown in Figs. 11, 12, 13 and 14. Groundwater samples before treatment showed seasonal variations in temperature, pH, color and turbidity, with many parameters exceeding the FAO-recommended limits for irrigation water quality. The greatest deterioration was generally observed during the summer season, especially for color and turbidity levels, which may have been due to increased evaporation, particulate suspension and microbial activity.

After treatment, the groundwater temperature was more stable, ranging between 23 and 24 °C in all the seasons, as shown in Figs. 11, 12, 13 and 14(a). The pH values were also slightly adjusted toward neutral conditions, ranging from 7.1 to 7.4, and remained well within the FAO permissible limits, as shown in Figs. 11, 12, 13 and 14 (b). The color levels also significantly decreased after the treatment. The color concentrations were reduced to 3–15 PtCo in the winter, 1–10 PtCo in the spring, 3–12 PtCo in the summer and 3–12 PtCo in the autumn, with most of the groundwater samples conforming to the FAO permissible limit of 15 PtCo, as shown in Figs. 11, 12, 13 and 14 (c).

Similarly, turbidity levels substantially decreased after treatment across all the seasons. The turbidity values decreased to 1–18 NTU in the winter, 6–17 NTU in the spring, 7–17 NTU in the summer and 1–15 NTU in the autumn, as illustrated in Figs. 11, 12, 13 and 14(d). The greatest reduction in turbidity was observed in the summer season, with the greatest deterioration in the pretreatment values. Nevertheless, despite these reductions, the posttreatment turbidity values frequently remained above the FAO permissible limit of 5 NTU-, particularly during spring and summer, when all samples exceeded the threshold, indicating that the filtration system alone did not consistently achieve the recommended turbidity level and that an additional polishing or filtration stage may be needed. In general, the results demonstrate that the AgNP/AC nanocomposite filtration system improved the physical quality of the groundwater, stabilized the temperature, adjusted the pH toward neutrality, and brought the color within permissible limits; however, residual turbidity indicates that a complementary filtration step is needed before full compliance with the irrigation turbidity requirements can be achieved.

### Trace elements and nutrient indicators

**Fig. 15:**
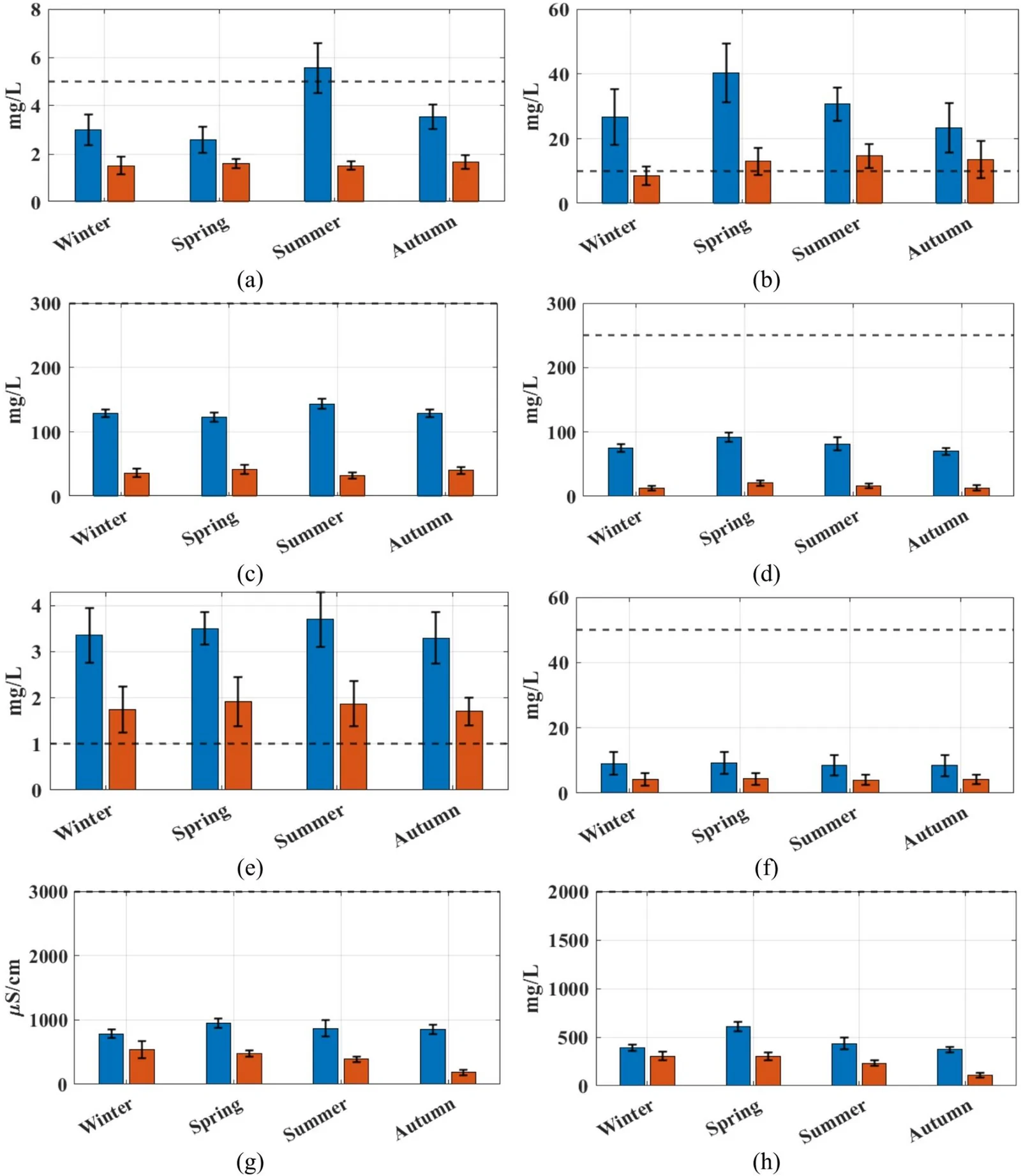

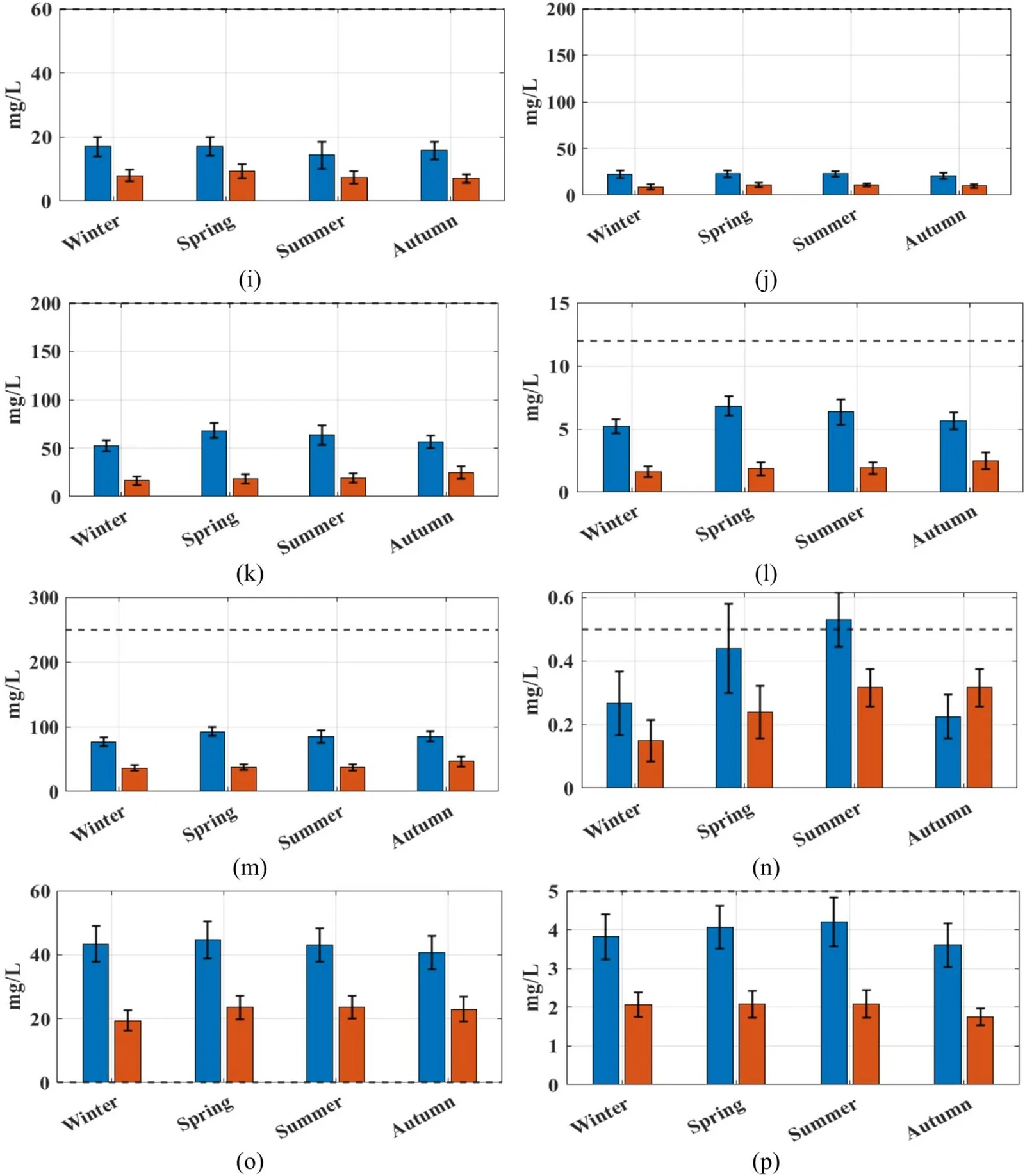

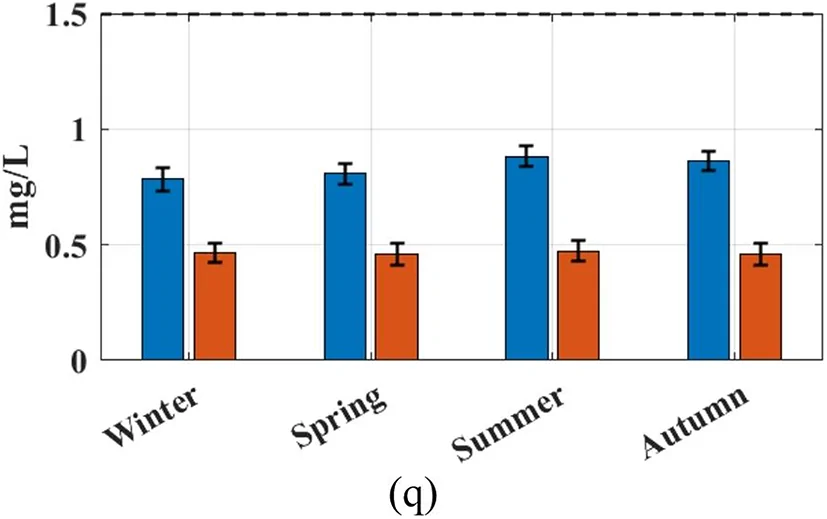
Variation of groundwater quality parameters before and after treatment (**a**) DO in mg/L. , (**b**) Acidity, (**c**) Alkalinity, (**d**) SO₄²^-^, (**e**) NO₂^-^, (**f**) NO₃^-^, (**g**) EC, (**h**) TDS, (**i**) Mg²⁺, (**j**) Ca²⁺, (**k**) Na⁺, (**l**) K⁺, (**m**) Cl^-^, (n) Silica, (**o**) Cu²⁺, (**p**) Fe²⁺, and (**q**) F^-^.

The analysis of groundwater quality parameters before treatment revealed significant differences among wells and seasons. These variations are summarized in Fig. 15(a–q) using the seasonal mean ± standard deviation values calculated from the 13 wells. Some parameters exceeded or did not satisfy the adopted guideline limits. The DO concentrations were relatively low to moderate, ranging from 1.64 to 6.90 mg/L before treatment, as shown in Fig. 15(a). Low DO values were recorded in all the wells during winter, spring and autumn. In summer, however, the lowest DO values were recorded mainly in wells W4–W6. The acidity and alkalinity values were moderate to high, and an alkalinity of 156 mg/L as presented in Fig. 15(b, c). The concentration of SO_4_^−2^ ranged from 56.7 to 98.6 mg/L, and the concentration of NO_3_^−^ reached 15.3 mg/L, which could be due to anthropogenic activities such as agricultural activities (Fig. 15(d–f)). NO₂^-^ represented one of the main pretreatment concerns, as it exceeded the adopted limit in all wells during the four seasons. The EC values reached a maximum of 1111 µS/cm, recorded in summer at sampling point W1, while the average EC before treatment was approximately 864 µS/cm, as shown in Fig. 15(g). The TDS concentration also peaked at 677.8 mg/L, indicating moderate salinity conditions, as illustrated in Fig. 15(h). The concentrations of the major cations Mg²⁺, Ca²⁺, Na⁺, and K⁺ also greatly varied as illustrated in Fig. 15(i–l), with maximum values of 21.04, 28.00, 83.86, and 8.39 mg/L, respectively, possibly due to mineral dissolution and ion-exchange. Cl^-^ ranged from 59.08 to 98.20 mg/L, as illustrated in Fig. 15(m). Trace and pollution-related parameters such as silica, Cu²⁺, Fe²⁺, and F^-^ also showed variability, as illustrated in Fig. 15(n–q). Cu²⁺ exceeded the adopted limit in all wells before treatment in all four seasons, indicating a persistent site-wide concern that requires further analysis. Fe²⁺ was generally close to or below the recommended limit; however, in the summer wells W2 and W12, it reached 5.10 mg/L, which is slightly above the adopted limit of 5 mg/L.

The post treatment mean values presented in Fig. 15(a–q) further suggest a notable increase in groundwater quality for most of the parameters during different seasons after treatment with the AgNP/AC nanocomposite system. The level of DO decreased from 0.83 to 2.20 mg/L, as shown in Fig. 15(a). Although this decrease was not a treatment target, post treatment DO values remained low in all wells during all the seasons, indicating the need for reaeration or posttreatment oxygen recovery when required for irrigation practices. The acidity and alkalinity also decreased, with the alkalinity decreasing to 26–58 mg/L (Fig. 15b, c). Sulphate, nitrite, and nitrate were markedly reduced after treatment. SO₄²^-^ decreased to 7.8–31.0 mg/L, NO₂^-^ decreased to 0.79–2.60 mg/L, and NO₃^-^ remained below 7.5 mg/L, as shown in Fig. 15(d–f). Despite the reduction in NO₂^-^, it remained above the adopted limit in most treated samples: W1–W7 and W9–W13 during winter and spring and all wells during summer and autumn. Only W8 in the winter and spring was below the adopted NO₂^-^ limit. The salinity-related parameters also improved, with the EC decreasing to 141–890 µS/cm and the TDS decreasing to 84.6–380.4 mg/L after treatment, as shown in Fig. 15(g, h). The concentrations of the major cations Mg²⁺, Ca²⁺, Na⁺, and K⁺ decreased to 3.87–13.10 mg/L, 5.61–14.90 mg/L, 8.81–34.41 mg/L, and 0.881–3.441 mg/L, respectively, as shown in Fig. 15(i–l). The concentration of chloride also decreased to 27.4–57.4 mg/L, as shown in Fig. 15(m). Most trace and pollution-related parameters improved after treatment, as shown in Fig. 15(n–q); for example, Fe²⁺ remained below 5 mg/L in all treated samples. In contrast, the concentration of copper remained elevated despite treatment, decreasing from a pretreatment range of 29.4–52.6 mg/L to 13.7–28.3 mg/L after treatment (Fig. 15(o)). Accordingly, Cu²⁺ remained above the adopted limit in all wells after treatment across the four seasons, suggesting that the proposed filtration unit reduced Cu²⁺ but was insufficient to achieve full compliance with this parameter. The fluoride concentration remained within the permissible limits after treatment, which ranged from 0.35 to 0.53 mg/L, as shown in Fig. 15.

### Microbiological Assessment

**Fig. 16:**
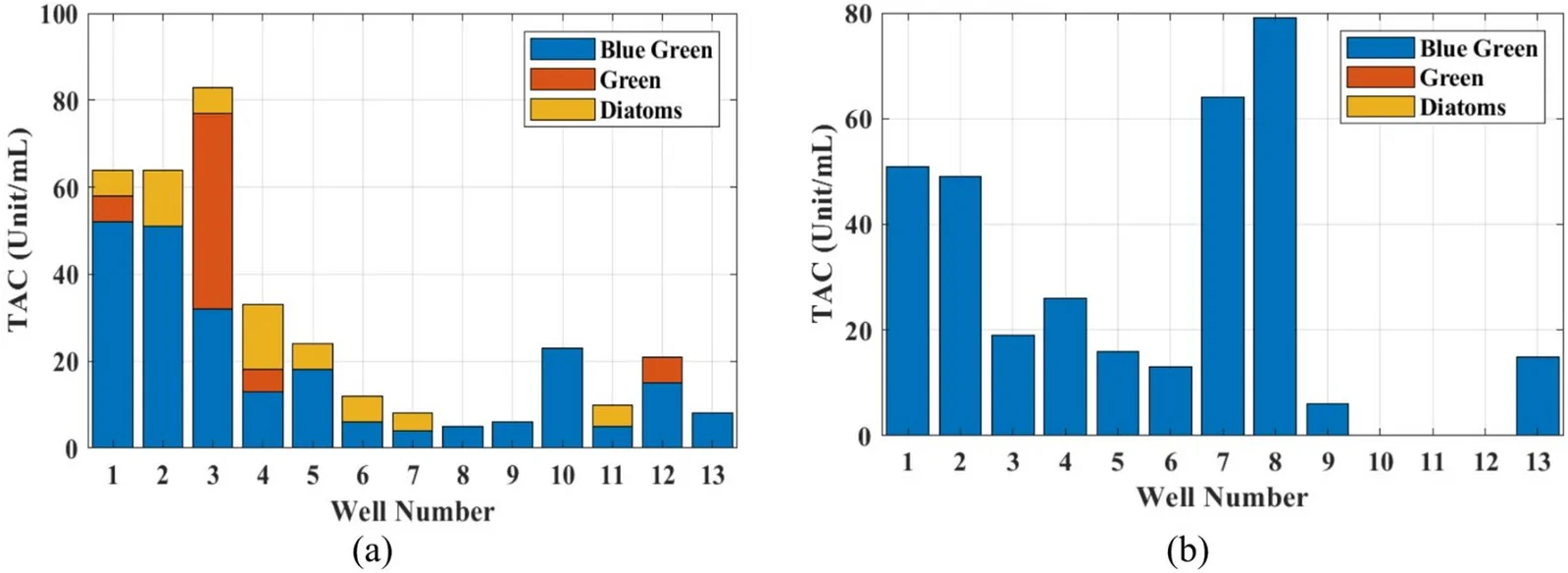
Before treatment microbiological analysis (**a**) spring and (**b**) summer.

**Fig. 17:**
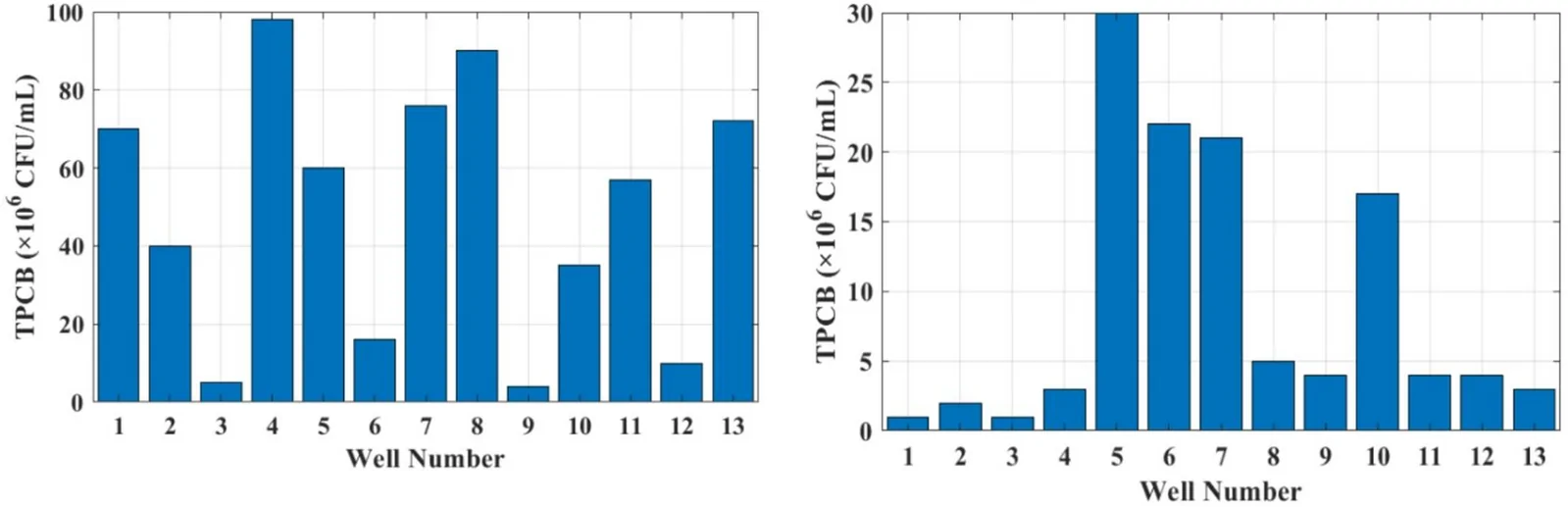
Temporal and spatial distribution of TPC during the study period (**a**) spring and (**b**) summer.

**Fig. 18:**
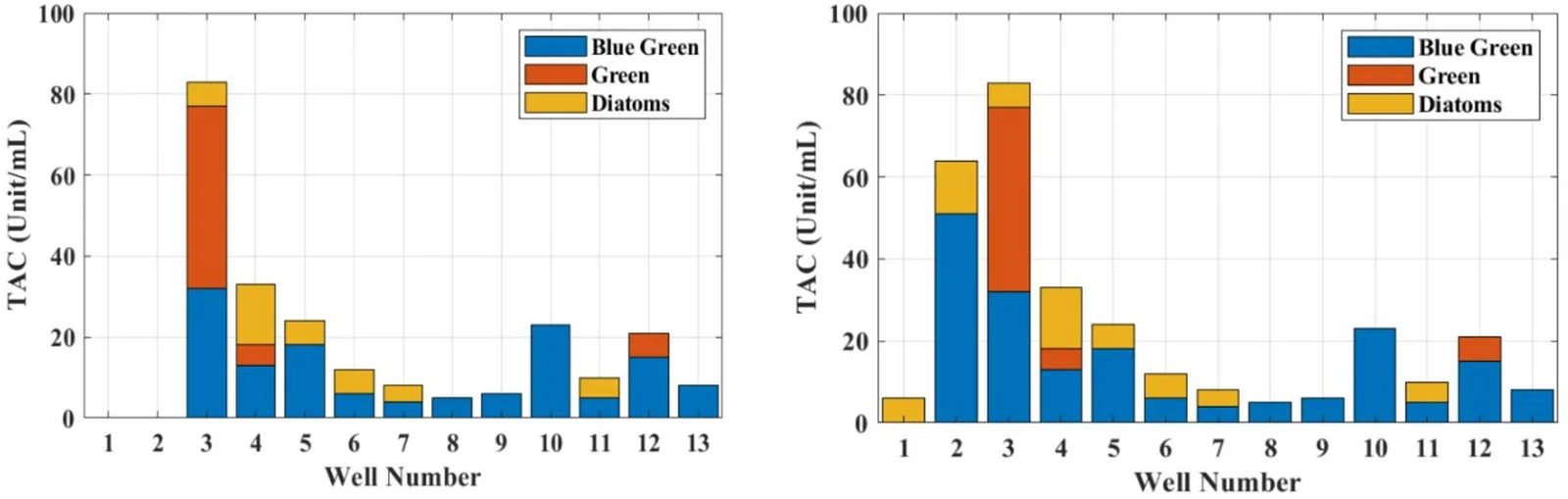
Post treatment microbiological analysis (**a**) spring and (**b**) summer.

**Fig. 19:**
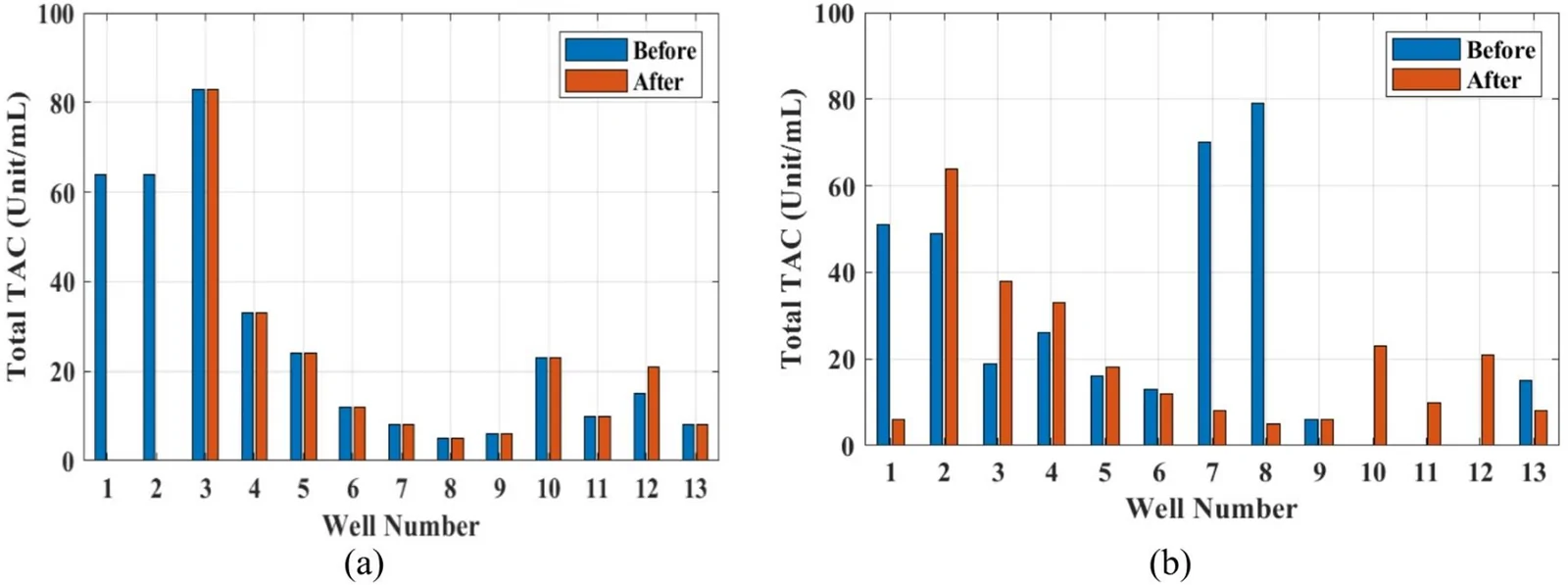
Total algae count before and after treatment (**a**) spring and (**b**) summer.

For the microbiological assessment, it should be noted that the total algal count (TAC) and total plate count (TPC) analyses were conducted only for the spring and summer seasons, which represent the warm-season periods of elevated temperature and nutrient availability associated with the greatest algal and bacterial proliferation. Accordingly, all microbiological results reported in this section and in Figs. (16–19) correspond to these two seasons.

The pretreatment microbiological assessment of the 13 boreholes revealed seasonal and spatial variations in both phytoplankton composition and bacterial density, as shown in Figs. 16 and 17. The phytoplankton community comprises three principal algal groups: blue–green algae (cyanobacteria), green algae, and diatoms. Cyanobacteria were the dominant algal group in most wells before treatment, with Anacystis being the most frequently isolated species. In spring (Fig. 16(a)), several wells still had algal populations with cyanobacteria, especially Anacystis and Oscillatoria, still dominant in the phytoplankton community. These species are commonly associated with nutrient-enriched environments. Compared with cyanobacteria, green algae and diatoms were also detected, although generally at lower abundances. As shown in Fig. 16(b) for summer; cyanobacteria remained the dominant group, mainly in wells 7 and 8, where high TAC values were generally linked to blue–green algae. The decrease in the occurrence of green algae and diatoms in some wells may suggest seasonal changes in the phytoplankton community under warmer environmental conditions. Using TPC test, bacterial contamination results before treatment also showed significant seasonal variation as depicted in Fig. 17, with substantial bacterial contamination in spring as TPC values ranged from 4 × 10⁶ to 98 × 10⁶ CFU/mL, especially in wells 4, 8, and 7 as presented in Fig. 17(a). During the summer season as shown by Fig. 17(b), the TPC values ranged from 1 × 10⁶ to 30 × 10⁶ CFU/mL, with elevated counts observed in wells 5, 6, and 7. This increased bacterial count may be associated with favourable environmental conditions, increased nutrient availability, and enhanced microbial activity during spring and summer.

In terms of the post-treatment results, algae presence was still detectable in some wells, as presented in Figs. 18 and 19, although the distribution patterns became more localized. After treatment, TAC values were positive in wells 3, 4, 5, and 10, whereas cyanobacteria (Anacystis) were still the major phytoplankton in most of the positive samples. Diatoms (Navicula, Cyclotella) and green algae (Chlorella) were also present in some wells indicating that phytoplankton growth was inhibited but not completely eliminated. In summer (Fig. 18(b)), algae were still present after treatment, with the highest TAC values in wells 2 (64 U/mL), 3 (83 Unit/mL) and 4 (33 Unit/mL). Cyanobacteria remained the dominant phytoplankton, especially Anacystis. The continuous detection of Oscillatoria indicates that the treatment process reduced the abundance of algae but did not completely inhibit cyanobacterial growth during warmer seasons. Significant reductions in TAC were recorded in spring and summer, but complete algal removal was not consistently achieved.

The quantitative TAC results before and after treatment are presented in Fig. 19(a–b) for spring and summer, respectively. Treatment was most effective in the wells with the highest initial algal loads, reducing the TAC to below the detection limit in wells 1 and 2 during spring and markedly lowering it in wells 1, 7, and 8 during summer (e.g., from 70 -79 to 5 - 8 units/mL). Averaged across the thirteen wells, these changes corresponded to TAC reductions of approximately 34% in spring and 14% in summer. Algal removal was nonetheless incomplete and spatially variable. Several wells with low initial counts showed slight increases post-treatment, and cyanobacteria, particularly the pollution-tolerant genus Oscillatoria, remained detectable, indicating that the treatment process did not fully eliminate the algal community. From a bacterial standpoint, TPC remained detectable in several wells after treatment, especially wells 4, 7, 8, and 13, confirming that complete bacterial removal was not consistently achieved, especially during the warmer seasons. It is worth noting that, however, total coliform and fecal coliform concentrations remained below the detection limit of less than < 1 CFU/100 mL in most samples, satisfying the commonly adopted FAO/WHO irrigation criteria for fecal indicator bacteria. The treated groundwater may therefore be considered acceptable for irrigation reuse from a fecal-contamination perspective, although the persistence of TPC and residual algae indicates that the proposed AgNP/AC system should not be regarded as a stand-alone disinfection process and that complementary disinfection may be required where more stringent microbiological standards apply.

### Irrigation Index

**Fig. 20:**
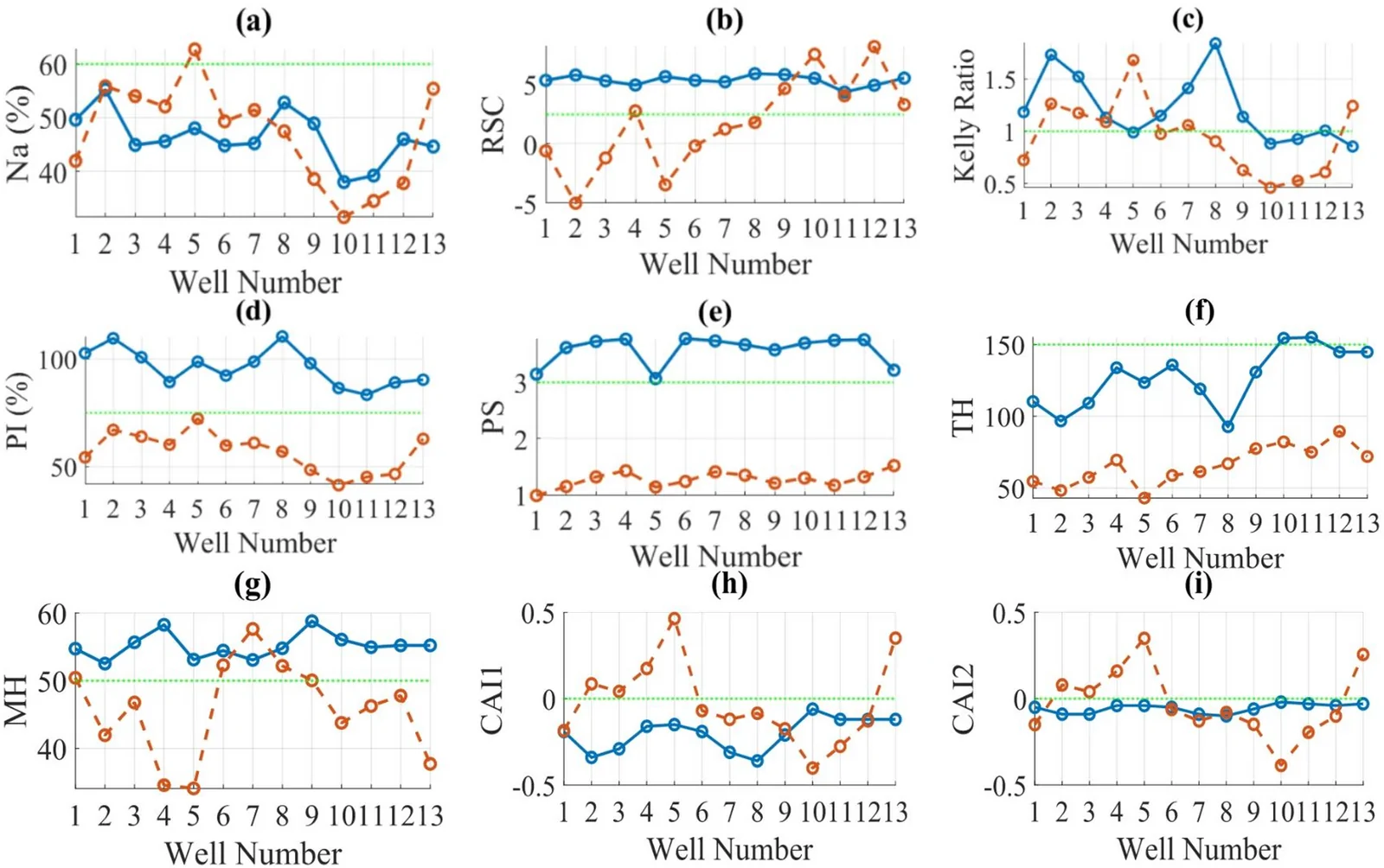
Variation of groundwater irrigation indices before and after treatment for spring: (**a**) Na%, (**b**) RSC, (**c**) KR, (**d**) PI, (**e**) PS, (**f**) TH, (**g**) MH, (**h**) CAI1, and (**i**) CAI2.

**Fig. 21:**
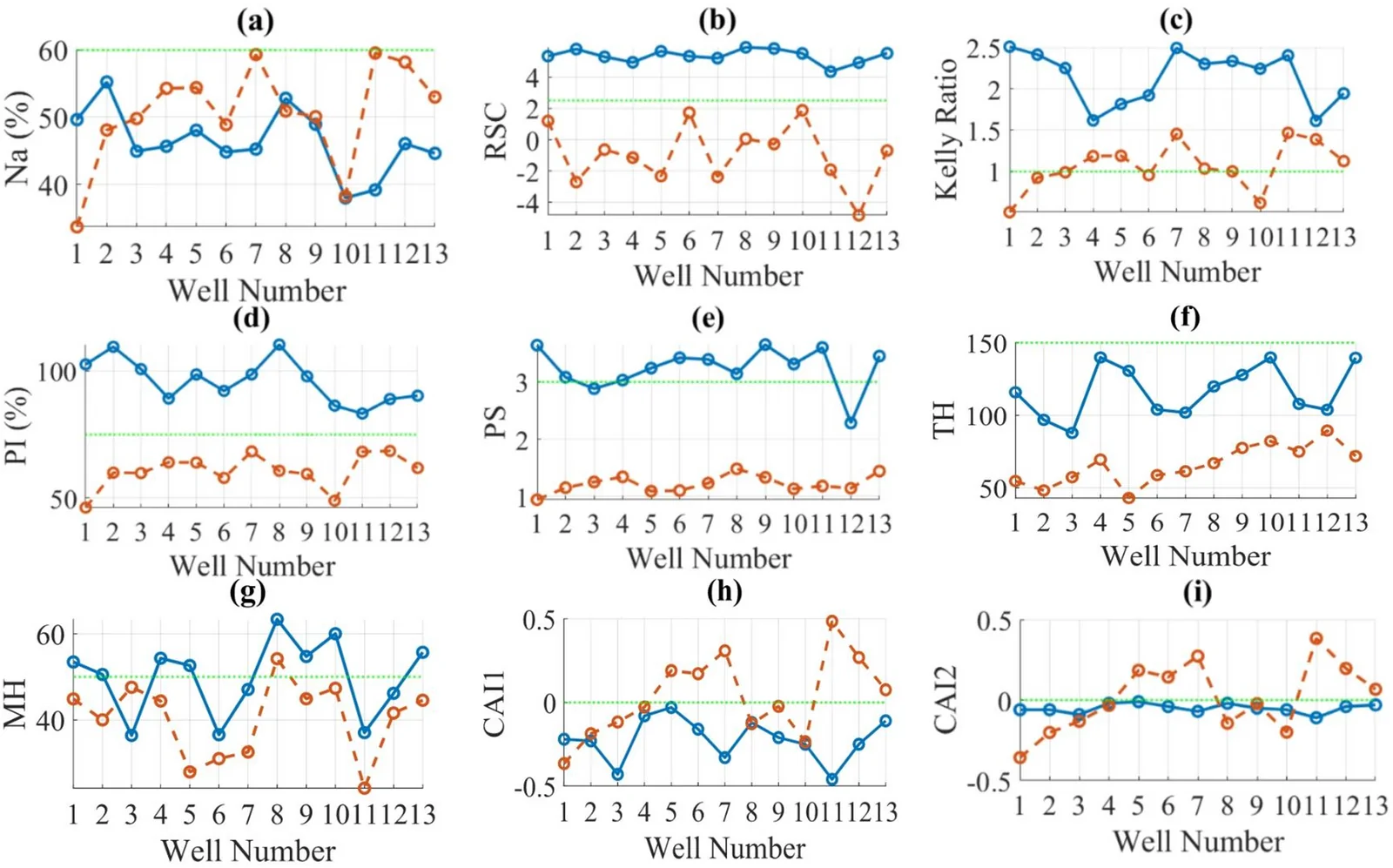
Variation of groundwater irrigation indices before and after treatment for Summer: (**a**) Na%, (**b**) RSC, (**c**) KR, (**d**) PI, (**e**) PS, (**f**) TH, (**g**) MH, (**h**) CAI1, and (**i**) CAI2.

**Fig. 22:**
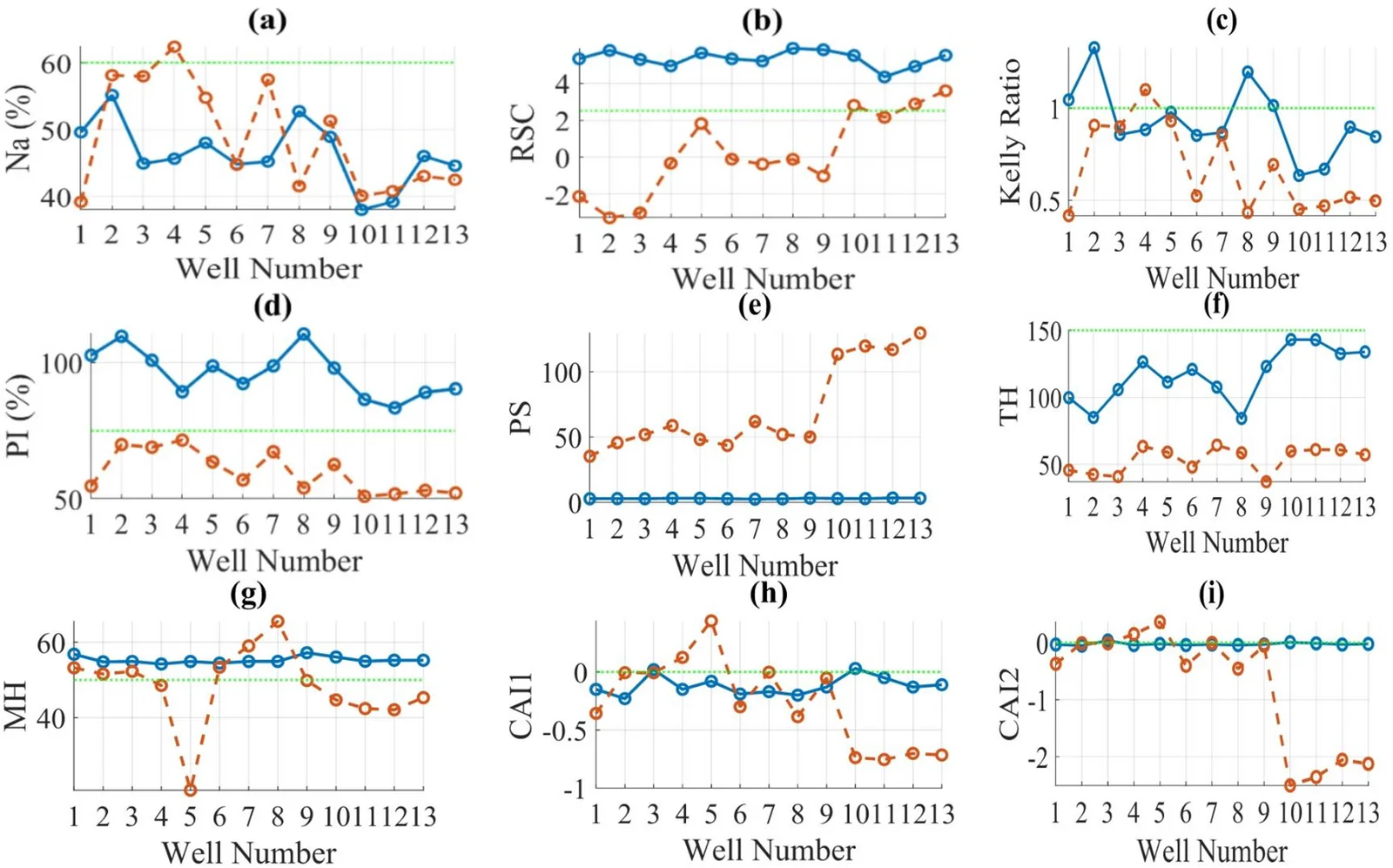
Variation of groundwater irrigation indices before and after treatment for winter: (**a**) Na%, (**b**) RSC, (**c**) KR, (**d**) PI, (**e**) PS, (**f**) TH, (**g**) MH, (**h**) CAI1, and (**i**) CAI2.

**Fig. 23:**
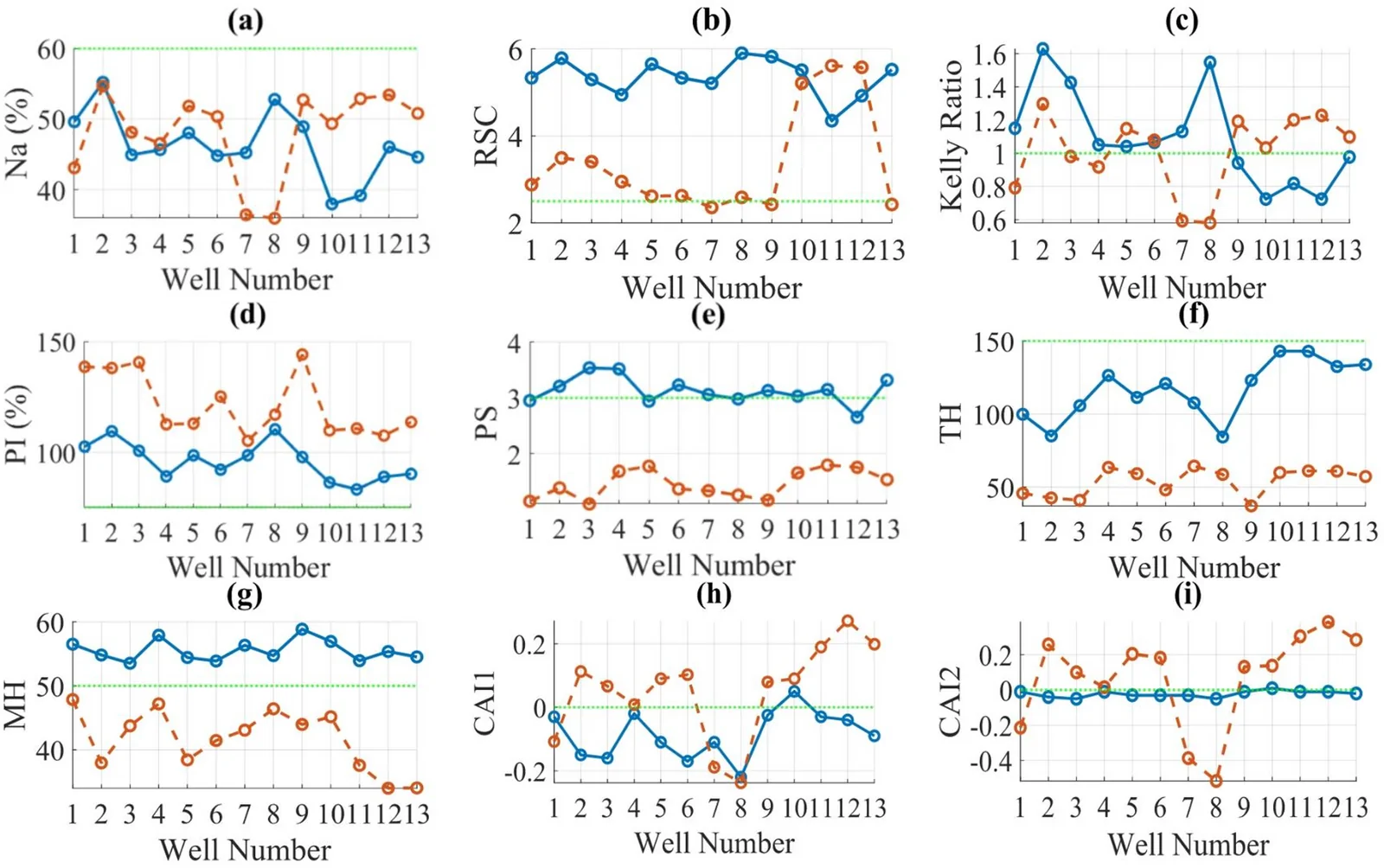
Variation of groundwater irrigation indices before and after treatment for autumn: (**a**) Na%, (**b**) RSC, (**c**) KR, (**d**) PI, (**e**) PS, (**f**) TH, (**g**) MH, (**h**) CAI1, and (**i**) CAI2.

Before treatment, the water quality of the groundwater used for irrigation, on the basis of the application of different indices, was found to show moderate to high levels of salinity, sodicity, and alkalinity hazards, with seasonal variations. The Na% values, which varied between 37.9 and 55.2% in winter, 38.0 and 55.2% in spring, 37.9 and 55.2% in summer, and 37.9 and 55.2% in autumn, were found to indicate moderate to high levels of sodium hazards, especially in summer, as illustrated in Figs. 20, 21, 22 and 23(a). Similarly, the KR, which was found to be above the safe limit of 1, varied between 0.63 and 1.33 in the winter, 0.85 and 1.84 in the spring, between 1.61 and 2.51 in the summer, and between 0.72 and 1.63 in the autumn, indicating that a large percentage of wells were not suitable for irrigation because of sodium dominance, as shown in Figs. 20, 21, 22 and 23(c). The RSC values, which varied between 4.35 and 5.90 meq/L in the winter, between the values of 4.35 and 5.90 meq/L in the spring, 4.35 and 5.90 meq/L in the summer, and 4.35 and 5.90 meq/L in the autumn, respectively, were found to indicate potential risks of alkalinity hazards, with most wells having values above 2.5 meq/L, which indicates potential soil alkalinity and permeability hazards, as presented in Figs. 20, 21, 22 and 23 (b). The permeability index values were found to be high, ranging between 83.25 and 110.32% in the winter, between 83.25 and 110.32% in the spring, 83.25 and 110.32% in the summer, and 83.25 and 110.32% in the autumn, as shown in Figs. 20, 21, 22 and 23 (d).

In terms of the salinity indicators, the PS levels were relatively low, ranging from 2.46 to 3.33, 3.06 to 3.77, 2.28 to 3.65, and 2.65 to 3.54, respectively, during the winter, spring, summer, and autumn seasons, as illustrated in Figs. 20, 21, 22 and 23 (e). Moreover, the TH levels were relatively high, ranging from 92.70 to 155.05, 92.70 to 155.05, 87.86 to 139.89, and 84.31 to 143.11, respectively, during the winter, spring, summer, and autumn seasons, as shown in Figs. 20, 21, 22 and 23 (f). These levels indicate high concentrations of calcium and magnesium ions. In terms of the MH, the MH levels were relatively high, ranging from approximately 52% to 57%, approximately 52% to 58%, 36% to 63%, and approximately 53% to 59% during the winter, spring, summer, and autumn, respectively, as presented in Figs. 20, 21, 22 and 23 (g). These levels exceeded the recommended limit of 50% and thus posed potential risks to soil quality. Finally, the CAI1 and CAI2 chloro-alkaline indices were predominantly negative during all the seasons, ranging from − 0.46 to 0.03 and from − 0.11 to 0.04, respectively, as illustrated in Figs. 20, 21, 22 and 23(h, i). These levels show the dominance of reverse ion exchange processes. In conclusion, the assessment of the groundwater prior to treatment revealed that the groundwater may be considered somewhat satisfactory, although there were potential irrigation constraints, especially in relation to the sodium hazard and alkalinity.

**Fig. 24:**
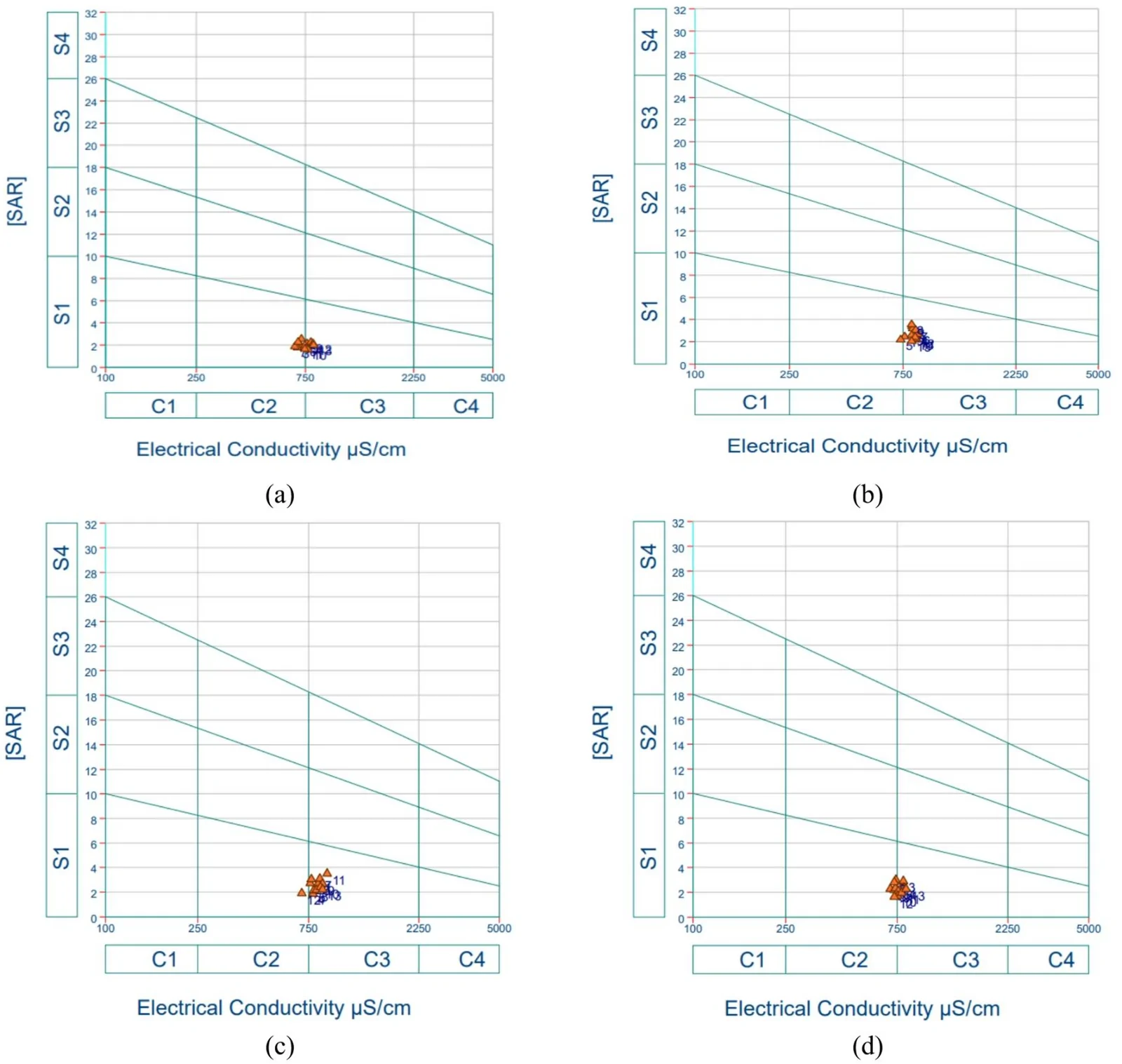
Classification of groundwater based on Salinity and Alkalinity Hazard (USSL Diagram) during (**a**) winter, (**b**) autumn, (**c**) spring and (**d**) summer seasons^60^.

With respect to the SAR values shown in Fig. 24(a–d), most of the groundwater samples for all the seasons tend to fall within the S1 class (low sodium hazard), with some fluctuations between wells and seasons. The grouping of the data points within the C2–S1 and C3–S1 classes implies that, although the salinity of the groundwater is moderate, the sodium hazard remains low and does not pose a significant risk to soil structure and permeability. It is also observed from Fig. 24(b) and Fig. 24(d) that the SAR values are slightly higher during the autumn and summer, which may be attributed to the effect of increased evaporation. Overall, the classification of SAR indicates that groundwater is generally suitable for irrigation purposes, provided that appropriate soil management practices are applied.

After the groundwater samples were treated with the AgNP/AC nanocomposite filtration system, the groundwater quality markedly improved, as indicated by all the irrigation indices, as clearly shown in Figs. 20, 21, 22 and 23. The Na% values ranged from 39.17 to 62.43% in the winter, 31.47–62.69% in the spring, 33.67–59.49% in the summer, and 34.00–63.24% in the autumn, as illustrated in Figs. 20, 21, 22 and 23(a). Although reductions were observed in several wells, the improvement in Na% was not uniform across all the samples.

The improvement in groundwater quality was also confirmed by the decrease in the Kelly ratio, which ranged from 0.64 to 1.66 in the winter, 0.46 to 1.68 in the spring, 0.51 to 1.47 in the summer, and 0.87 to 1.86 in the autumn, as shown in Figs. 20, 21, 22 and 23 (c).

On the other hand, the RSC values decreased significantly after the treatment process, changing to − 3.29 to 3.60 meq/L in the winter, − 5.00 to 8.18 meq/L in the spring, − 4.82 to 1.88 meq/L in the summer, and 1.08 to 12.23 meq/L in the autumn, as presented in Figs. 20, 21, 22 and 23 (b). Although some of the values are still slightly above the tolerable limit, the overall decrease in RSC values indicates better control of the concentration of carbonates and bicarbonates in the water. The PI values ranged from 50.86 to 71.44% in the winter, 41.31–72.19% in the spring, 46.31–68.54% in the summer, and 53.25–71.84% in the autumn, as shown in Figs. 20, 21, 22 and 23 (d). Although lower than the pretreatment values, these results remain within acceptable irrigation suitability classes and indicate more realistic soil permeability conditions, as shown in Figs. 20, 21, 22 and 23 (d).

In response to the reduction in chloride and sulphate, the PS values decreased after treatment, decreasing to 0.85–1.40 in the winter, 0.99–1.52 in the spring, 0.94–1.48 in the summer, and 1.09–1.79 in the autumn, as illustrated in Figs. 20, 21, 22 and 23 (e). Since all posttreatment PS values were well below the recommended threshold of 3, the treated groundwater was classified as suitable according to the PS criterion, confirming that the salinity hazard was effectively mitigated.

The TH values decreased significantly after the treatment process, as the values changed to 40.63–72.49 mg/L in winter, 42.97–89.49 mg/L in spring, 42.97–89.49 mg/L in summer, and between 37.16 and 64.42 mg/L in autumn, as shown in Figs. 20, 21, 22 and 23 (f). The values of MH have decreased significantly after the treatment process, as the values changed to 20.73%–65.54% in winter, 34.09%–57.64% in spring, 24.14%–54.21% in summer, and 34.00%–47.86% in autumn, as presented in Figs. 20, 21, 22 and 23 (g), as most of the values are below the critical value of 50%. Most samples became suitable according to the MH criterion, particularly during summer and autumn.

Furthermore, the values of CAI1 and CAI2 changed in a direction towards less negative or even positive values up to 0.48 and 0.38, respectively, as illustrated in Figs. 20, 21, 22 and 23 (h, i), which indicates a change in the hydrochemical processes and ion exchange behaviour.

### Statistical analysis

To confirm whether the observed seasonal differences in groundwater quality and the improvements recorded after treatment represent variation rather than sampling or analytical noise, the collected samples were subjected to formal statistical analysis. Each reported value is the mean of three independent field replicates collected per well in each season (156 samples in total) and is expressed with its standard deviation to convey measurement variability. Differences among the four seasons were examined using one-way analysis of variance (ANOVA), as presented in Table 5 for the pre-treatment samples; all tests were two-tailed, and *p* < 0.05 was considered statistically significant. As summarized in Table 5, most physicochemical parameters varied significantly across the four seasons. The salinity and ion-related parameters showed the strongest seasonal dependence, with EC, TDS, Na⁺, K⁺, Cl^-^, and SO₄²^-^ differing significantly among seasons (*p* ≤ 0.001), with the highest mean values consistently recorded during the spring and summer and the lowest during the winter and autumn periods. EC and TDS peaked in spring (950.15 ± 75.14 µS/cm and 608.04 ± 8.07 mg/L, respectively), whereas Na^-^ and K^-^ reached their maximum values in spring (68.27 ± 7.78 and 6.83 ± 0.78 mg/L, respectively) and summer (63.45 ± 10.08 and 6.35 ± 1.01 mg/L, respectively), and Cl^-^ and SO₄²^-^ followed the same pattern. Acidity, alkalinity, silica, and dissolved oxygen were likewise significantly season dependent (*p* < 0.0001), with the concentrations of silica and dissolved oxygen highest in the summer (0.531 ± 0.085 and 5.57 ± 1.04 mg/L, respectively) and the acidity highest in the spring (40.35 ± 9.07 mg/L). In contrast, pH, NO₂^-^, NO₃^-^, and Mg²⁺ did not significantly differ (*p* = 0.076, 0.236, 0.909, and 0.078, respectively). Overall, the mean concentrations of the major cations, anions, and trace metals were greater during the dry season than during the wet season, which is consistent with reduced rainfall dilution and enhanced evaporative concentration; the elevated levels may also reflect the seepage of wastewater into the aquifer and, given the coastal setting of the study area, possible seawater intrusion.

**Table 5:** Descriptive statistics of the physicochemical parameters of groundwater before treatment.

Parameter	Winter wet season			Spring dry season			Summer dry season			Autumn wet season			*p* value
	Min	Max	Mean ± SD	Min	Max	Mean ± SD	Min	Max	Mean ± SD	Min	Max	Mean ± SD	
DO	1.76	3.94	3.00 ± 0.64	1.64	3.50	2.59 ± 0.53	3.45	6.9	5.57 ± 1.04	2.8	4.6	3.55 ± 0.5	< 0.0001
Silica	0.12	0.451	0.266 ± 0.1	0.08	0.64	0.440 ± 0.140	0.39	0.66	0.531 ± 0.085	0.122	0.35	0.225 ± 0.07	< 0.0001
pH (no unit)	7.1	8.6	7.65 ± 0.38	7.4	8.0	7.66 ± 0.19	7.4	8.4	7.71 ± 0.27	7.3	7.6	7.46 ± 0.1	0.076
Acidity	16.8	40.6	26.74 ± 8.55	23.6	54.0	40.35 ± 9.07	22.6	38.8	30.74 ± 5.07	14.3	38.4	23.47 ± 7.56	< 0.0001
Alkalinity	123	142	129.15 ± 5.98	114	139	123.23 ± 7.41	131	156	143.85 ± 7.9	119	139	128.85 ± 5.73	< 0.0001
EC (µS/cm)	661	890	793.36 ± 61.52	802	1059	950.15 ± 75.14	608	1111	868.15 ± 125.64	712	987	854.0 ± 72.97	< 0.0001
TDS (mg/L)	330.5	445	396.68 ± 30.76	513.28	677.76	608.04 ± 8.07	304	555.5	434.08 ± 62.82	334.5	417.5	371.22 ± 25.75	0.001
SO_4_^2-^ (mg/L)	62.5	85.5	74.98 ± 6.56	78.4	98.6	92.23 ± 7.18	59.0	92.7	81.52 ± 10.18	56.7	75.8	69.72 ± 5.29	< 0.0001
NO_2_^-^ (mg/L)	2.67	4.59	3.35 ± 0.6	2.89	4.08	3.50 ± 0.35	2.74	4.66	3.69 ± 0.6	2.54	4.45	3.29 ± 0.56	0.236
NO_3_^-^ (mg/L)	5.30	15.10	9.08 ± 3.48	5.8	15.3	9.24 ± 3.44	5.1	14.9	8.55 ± 3.07	5.1	14.3	8.45 ± 3.27	0.909
CL^−^ (mg/L)	64.08	86.34	76.46 ± 6.62	79.36	98.0	92.56 ± 6.49	59.08	96.56	84.82 ± 10.17	69.08	98.2	85.32 ± 7.81	< 0.0001
Mg^2+^ (mg/L)	12.34	21.04	16.96 ± 2.94	12.34	21.04	17.05 ± 2.99	7.77	20.4	14.38 ± 4.24	11.21	19.8	15.76 ± 2.76	0.078
Ca^2+^ (mg/L)	16.8	28	22.65 ± 3.97	16.8	28.0	22.79 ± 3.51	17.61	27.22	23.03 ± 2.76	15.3	26.4	20.78 ± 3.5	0.004
Na^-^ (mg/L)	43.6	59.8	52.42 ± 5.51	56.1	78.4	68.27 ± 7.78	45.3	83.9	63.45 ± 10.08	44.1	69.4	56.52 ± 6.74	< 0.0001
K^-^ (mg/L)	4.36	5.98	5.24 ± 0.55	5.61	7.84	6.83 ± 0.78	4.53	8.39	6.35 ± 1.01	4.41	6.94	5.65 ± 0.67	< 0.0001
Cu (mg/L)	31.1	51.1	43.88 ± 5.64	31.9	52.6	44.59 ± 5.75	31.1	51.2	43.09 ± 5.26	29.4	47.8	40.71 ± 5.15	0.022
Fe (mg/L)	2.8	4.7	3.80 ± 0.59	3.01	4.8	4.06 ± 0.56	3.04	5.1	4.20 ± 0.63	2.5	4.5	3.60 ± 0.56	0.010

**Table 6:** Descriptive statistics of the physicochemical parameters of groundwater after treatment.

Parameter	Winter wet season			Spring dry season			Summer dry season			Autumn wet season			*p* value
	Min	Max	Mean ± SD	Min	Max	Mean ± SD	Min	Max	Mean ± SD	Min	Max	Mean ± SD	
DO	0.83	1.97	1.52 ± 0.36	1.30	1.90	1.62 ± 0.19	1.30	1.90	1.52 ± 0.18	1.38	2.20	1.67 ± 0.30	0.418
Silica	0.064	0.273	0.149 ± 0.066	0.020	0.340	0.239 ± 0.083	0.240	0.400	0.316 ± 0.059	0.240	0.400	0.316 ± 0.059	< 0.0001
pH (no unit)	7.1	7.4	7.25 ± 0.11	7.1	7.4	7.22 ± 0.09	7.1	7.4	7.25 ± 0.11	7.1	7.4	7.25 ± 0.11	0.787
Acidity	4.80	14.60	8.77 ± 2.83	8.20	19.40	13.19 ± 4.14	9.30	20.10	14.82 ± 3.72	6.30	28.40	13.75 ± 5.66	0.003
Alkalinity	29.00	53.00	36.85 ± 6.80	34.00	58.00	42.15 ± 6.82	26.00	41.00	32.46 ± 4.56	32.00	47.00	40.77 ± 5.45	0.001
EC (µS/cm)	314.0	890.0	537.2 ± 128.7	406.0	560.0	475.1 ± 51.37	314.0	458.0	388.8 ± 45.11	141.0	261.0	183.8 ± 42.89	< 0.0001
TDS (mg/L)	188.4	380.4	306.2 ± 43.82	246.0	370.0	302.3 ± 40.00	188.4	274.8	233.3 ± 27.07	84.60	156.6	110.3 ± 25.68	< 0.0001
SO_4_^2-^ (mg/L)	7.80	19.40	12.80 ± 3.74	14.70	31.00	20.51 ± 4.12	9.80	21.40	16.21 ± 3.71	7.84	21.20	13.14 ± 4.38	< 0.0001
NO_2_^-^ (mg/L)	0.79	2.51	1.74 ± 0.50	0.96	2.60	1.91 ± 0.53	1.10	2.45	1.87 ± 0.49	1.28	2.32	1.70 ± 0.30	0.605
NO_3_- (mg/L)	1.23	7.40	4.26 ± 1.86	1.27	7.48	4.34 ± 1.86	1.30	7.50	4.02 ± 1.60	2.34	6.60	4.10 ± 1.44	0.961
CL^-^ (mg/L)	27.40	42.40	36.47 ± 4.37	29.30	43.10	37.54 ± 4.17	29.60	45.10	37.08 ± 4.67	34.70	57.40	46.58 ± 7.70	< 0.0001
Mg^2+^ (mg/L)	3.87	10.66	7.98 ± 1.78	4.81	13.10	9.32 ± 2.27	4.20	10.30	7.38 ± 1.95	5.10	9.20	7.04 ± 1.34	0.016
Ca^2+^ (mg/L)	5.61	14.80	8.84 ± 3.05	7.60	14.90	11.04 ± 2.75	8.70	13.80	10.78 ± 1.61	6.50	13.20	9.98 ± 2.19	0.111
Na^+^ (mg/L)	8.81	23.24	16.30 ± 4.37	11.79	28.76	18.36 ± 5.20	9.39	25.55	19.10 ± 4.55	15.70	34.41	24.76 ± 6.52	0.001
K^+^ (mg/L)	0.88	2.32	1.63 ± 0.44	1.18	2.88	1.84 ± 0.52	0.94	2.56	1.91 ± 0.46	1.57	3.44	2.48 ± 0.65	0.001
Cu (mg/L)	13.80	25.10	19.42 ± 3.28	15.70	28.30	23.50 ± 3.74	16.30	27.60	23.68 ± 3.59	13.70	27.30	22.97 ± 3.92	0.013
Fe (mg/L)	1.63	2.70	2.06 ± 0.32	1.70	2.80	2.08 ± 0.34	1.60	2.90	2.09 ± 0.36	1.20	1.97	1.74 ± 0.22	0.017

Following treatment, the same statistical framework was applied to the posttreatment dataset, as presented in Table 6. Statistically significant seasonal differences persisted for most parameters, including EC, TDS, SO₄²^-^, Cl^-^, Na⁺, K⁺, Mg²⁺, acidity, alkalinity, silica, Cu, and Fe (*p* < 0.05), indicating that the seasonal influence on groundwater chemistry was attenuated but not eliminated by filtration. In contrast, the pH, DO, NO₂^-^, NO₃^-^, and Ca²⁺ did not significantly change seasonally after treatment (*p* > 0.05). The posttreatment mean concentrations of the great majority of parameters fell well within the FAO (1994) irrigation limits across all four seasons; EC (183.8–537.2 µS/cm), TDS (110.3–306.2 mg/L), Na⁺ (16.3–24.8 mg/L), Cl^-^ (36.5–46.6 mg/L), alkalinity (32.5–42.2 mg/L), SO₄²^-^ (12.8–20.5 mg/L), silica (0.15–0.32 mg/L), and Fe (1.74–2.09 mg/L) all remained below their respective guideline values. Three parameters, however, remained above their limits after treatment: nitrite (NO₂^-^, 1.70–1.91 mg/L against a limit of 1 mg/L) exceeded the guideline in all the seasons; acidity (8.8–14.8 mg/L against 10 mg/L) exceeded it in the spring, summer, and autumn seasons; and copper (19.4–23.7 mg/L) remained far above the 0.2 mg/L limit, reinforcing the earlier indication of a localized copper source. The dissolved oxygen concentration remained low (1.52–1.67 mg/L) and did not significantly differ across seasons (*p* = 0.418). Overall, these results confirm that although the treatment system produced consistent improvements across all the seasons, a small number of parameters, most notably copper, nitrite, and acidity, require additional or dedicated polishing before full compliance with the irrigation guidelines can be achieved.

## Discussion

A comparative assessment of groundwater quality before and after treatment verified the efficiency of the developed AgNP/AC nanocomposite filtration system in enhancing the suitability of groundwater as an irrigation water source during different seasons. Tables 7 and 8 summarize changes in major dissolved ions and physicochemical water-quality parameters. As shown in Tables (7–8), the proposed system reduced most monitored groundwater quality parameters across the 13 wells and 4 seasons, with average reductions ranging from 30% to 80%. The greatest improvement was observed in the salinity-related parameters, as indicated by the EC decreasing by 54.2% and the TDS decreasing by 47.3%. Both values remained below the FAO irrigation limits of 3000 µS/cm and 2000 mg/L, respectively, indicating a clear decrease in the dissolved ionic load after treatment.

Additionally, major ions also decreased, with SO₄²^-^ showing a significant 80.3% removal among all the measured ions. Na⁺ and K⁺ followed, with a removal efficiency of 67.4%, whereas Ca²⁺, Cl^-^, and Mg²⁺ decreased by 54.4%, 53.5%, and 50.5%, respectively.

Similarly, the concentration of NO_3_^-^ decreased by 52.6%, remaining below 50 mg/L as per the FAO guidelines, whereas the concentration of NO₂^-^ decreased by 47.8%, from 3.46 to 1.81 mg/L, slightly above the adopted FAO guidelines. Posttreatment results revealed that the proposed framework also reduced several pH-related constituents. Alkalinity and acidity decreased by 71.0% and 58.3%, respectively. Silica showed a more moderate reduction of 30.2%. The fluoride concentration decreased by 44.3%, reaching 0.46 mg/L, which is below the adopted FAO limit of 1.5 mg/L.

With respect to trace metals, the concentration of Fe decreased by 49.2% and remained below the adopted FAO limit of 5 mg/L after treatment. For the Cu²⁺ values, however, both the pre- and posttreatment results indicate possible contamination in the area, which requires further investigation.

Notably, in addition to the observed improvements in chemical water quality, the treated water exhibited a marked reduction in DO. The DO concentration of the treated water markedly decreased from 3.76 to 1.59 mg/L. This reduction is not a treatment objective but a consequence of water passing through the packed activated-carbon/Ag-AC column, in which dissolved oxygen is consumed by adsorption onto the high-surface-area carbon, by surface redox interactions, and through the removal of oxygen-demanding organic constituents. Although a residual DO concentration is 1.59 mg/L, its practical impact on irrigation is expected to be limited. Under conventional surface or sprinkler application, the treated water is rapidly re-aerated upon contact with the atmosphere and the soil surface; thus, the low-DO state is unlikely to persist into the root zone of well-drained, structurally porous soils, where oxygen availability is controlled mainly by soil–air exchange rather than by the dissolved oxygen content of the applied irrigation water.

**Table 7:** Summary of major dissolved ions before and after treatment.

Parameter	Average Before	Average After	Average Reduction (%)	FAO Limit
Ca²⁺ (mg/L)	22.31	10.16	↓54.4	200
Mg²⁺ (mg/L)	16.03	7.93	↓50.5	60
Na⁺ (mg/L)	60.16	19.63	↓67.4	200
K⁺ (mg/L)	6.02	1.96	↓67.4	12
Cl^-^ (mg/L)	84.79	39.42	↓53.5	250
SO₄²^-^ (mg/L)	80.10	15.67	↓80.3	250
Cu²⁺ (mg/L)	42.94	22.43	↓47.9	0.2

**Table 8:** Summary of physiochemical parameters before and after treatment.

Parameter	Average Before	Average After	Average Reduction (%)	FAO Limit
Acidity (mg/L)	30.32	12.63	↓58.3	10
Alkalinity (mg/L)	131.27	38.06	↓71.0	300
NO₂^-^ (mg/L)	3.46	1.81	↓47.8	1
NO₃^-^ (mg/L)	8.83	4.18	↓52.6	50
EC (µS/cm)	864.24	396.23	↓54.2	3000
TDS (mg/L)	451.42	238.02	↓47.3	2000
Silica (mg/L)	0.37	0.26	↓30.2	0.5
Fe (mg/L)	3.92	1.99	↓49.2	5
F^-^ (mg/L)	0.83	0.46	↓44.3	1.5

↑: increase, ↓: decrease.

Table 9 summarizes the average values of the irrigation suitability indices before and after treatment. The results demonstrate that the AgNP/AC filtration system significantly improved several groundwater quality indicators associated with irrigation use. Substantial decreases were observed in RSC (71.69%), PS (60.40%), KR (21.71%), TH (49.76%), and MH (16.73%), indicating decreases in carbonate and salinity hazards, sodium-related risks, hardness, and magnesium hazards. These improvements are particularly important for reducing soil permeability deterioration, alkalinity hazards, and long-term soil structure degradation. The treatment process also resulted in a shift in CAI1 and CAI2 values toward less negative and, in some cases, positive values, suggesting modifications in ion exchange processes and groundwater hydrochemistry after filtration. Furthermore, the SAR values remained within the excellent irrigation class before and after treatment, confirming the generally low sodium absorption hazard of the investigated groundwater, where the treated samples remained within the S1 category on the USSL diagram, indicating a low risk of sodium and a limited likelihood of sodium-induced soil dispersion or permeability reduction.

However, not all indices improved. The Na% values showed only minor overall increases, indicating that, compared with reductions in hardness and carbonate-related parameters, the removal of sodium was limited. In contrast, PS decreased by 60.4% after treatment and fell below the recommended threshold in all samples, indicating that the salinity hazard was effectively mitigated. These findings suggest that while the filtration system effectively reduced several irrigation hazards, including salinity, residual sodium-related concerns may still persist and require further investigation. Therefore, the treated groundwater should be evaluated using multiple irrigation indices rather than relying on a single parameter.

The ion reductions after treatment reflects the combined action of the dual action of the AgNPs/AC. Activated carbon, on its owen, provides a high surface area and oxygen-containing functional groups, which has been confirmed by FTIR, as the water is buffered toward the near-neutral treated pH (~ 7.25), through promoting electrostatic attraction and ion exchange of the major cations (Na⁺, K⁺, Ca²⁺, Mg²⁺), leading to 50–67% removal efficiency^20^. As for antimicrobial activity, the carbon physically captures cells, algae, and organic matter but is not biocidal, whereas the immobilized silver nanoparticles inactivate microorganisms through Ag⁺ release, reactive-oxygen-species generation, and membrane and enzyme disruption^27,42,74^; the residual microbial counts in spring–summer are consistent with chloride (still 27–57 mg/L after treatment) forming AgCl and lowering bioavailable free Ag⁺. Regarding seasonal variation, in the spring–summer dry seasons lower dilution and higher evaporation raised salinity, ionic strength, turbidity, and microbial load, increasing competition for a fixed number of adsorption sites and lowering fractional removal, while higher temperatures shifted adsorption equilibria and increased disinfection demand.

**Table 9:** Summary of irrigation suitability indices before and after treatment.

Irrigation Indices	Average Before	Average After	Change (%)
Na (%)	47.92	48.28	↑ 0.75
SAR	2.66	1.90	↓ 28.6
RSC (meq/L)	5.37	1.52	↓ 71.69
KR	1.29	1.01	↓ 21.71
PI (%)	98.89	62.97	↓ 36.32
PS (meq/L)	3.22	1.28	↓60.25
TH (mg/L)	122.67	61.63	↓ 49.76
MH (%)	53.81	44.81	↓ 16.73
CAI1	−0.14	+ 0.02	Δ = +0.16
CAI2	−0.04	−0.02	Δ = +0.02

↑: increase, ↓: decrease, Δ indicates the absolute difference between the average after- and before-treatment values.

Although the AgNP/AC filtration system significantly improved several groundwater quality and irrigation suitability indicators, the potential environmental implications associated with the use of silver nanoparticles should also be considered. Since the AgNPs were immobilized on the activated carbon support, nanoparticle mobility is expected to decrease; however, prolonged operation may result in limited silver ion release or nanoparticle leaching. Therefore, further investigations are needed to evaluate the long-term environmental behavior of the filtration medium under continuous irrigation applications.

From a microbiological perspective, treatment effectiveness exhibited clear seasonal variability. Reductions in the TAC were observed during spring and summer, with average decreases of 34.40% and 14%, respectively, as shown in Table 10. Post treatment TAC was not assessed for the winter and autumn campaigns; accordingly, treatment-induced changes in algal abundance during these two seasons could not be quantified and are not reported. Furthermore, although reductions in algal abundance were achieved at several locations, elevated TPC values persisted in some spring and summer samples, indicating that the AgNP/AC filtration system provided partial microbial control rather than complete disinfection. Notably, the TPC represents the overall microbial load and does not necessarily indicate the presence of pathogenic microorganisms. Therefore, while the developed treatment system substantially improved the physicochemical characteristics and irrigation suitability of the groundwater, its microbiological performance was season dependent. Additional optimization and complementary disinfection processes may therefore be needed where enhanced microbial removal is necessary for long-term irrigation reuse and water quality management.

**Table 10:** Seasonal variation in TAC before and after treatment.

Season	Average TAC Before Treatment (Unit/mL)	Average TAC After Treatment (Unit/mL)	Reduction (%)
Winter	74.46	ND	—
Spring	27.31	17.92	34.40
Summer	26.46	22.85	14
Autumn	1.85	ND	—

ND = not determined. Posttreatment total algal count was analysed only for the spring and summer campaigns; winter and autumn posttreatment samples were not assessed for TAC.

### Comparison with related work

To provide a comprehensive assessment of the irrigation suitability of the treated groundwater, the present results were compared with **(i) recent groundwater quality assessment studies conducted in Egypt** and **(ii) groundwater treatment studies employing activated carbon-based composite filtration systems**. The former provides a benchmark for evaluating the hydrochemical characteristics and irrigation suitability classifications of groundwater, whereas the latter enables assessment of the treatment performance achieved in terms of improving water quality and irrigation suitability.

- **Comparison with recent groundwater quality assessment studies conducted in Egypt**:

Before comparison, the calculated irrigation indices were classified according to internationally recognized irrigation water quality criteria. The adopted classification limits for SAR, RSC, Na%, KR, PI, MH, PS, and TH are based on widely accepted irrigation water quality guidelines and the FAO irrigation water quality standards presented in^75,76^. Table 11 summarizes the percentage of groundwater samples falling within each suitability category after treatment for all the seasons, illustrating the proportion of wells classified as excellent, good, permissible, doubtful, suitable, or unsuitable according to each irrigation index. Accordingly, the present findings were compared with those of four recent groundwater quality assessment studies conducted in Egypt, which are listed in Table 1^52,53,56,58^. These studies were selected because they employed the same irrigation suitability indices used in the current work, including SAR, RSC, KR, PI, MH, and Na%, and investigated groundwater resources under comparable hydrogeological and climatic conditions. Furthermore, these studies represent recent applications of irrigation water quality assessment frameworks in arid and semiarid regions and therefore provide a suitable benchmark for evaluating the effectiveness of the proposed AgNP/AC treatment system.

An assessment of irrigation suitability after treatment revealed that the AgNP/AC nanocomposite filtration system improved several key irrigation indices, although some limitations remained. As shown in Table 9, all the groundwater samples were classified within the excellent SAR category (SAR < 10) across all the seasons, indicating a consistently low sodium adsorption hazard. Similar findings were reported by^52,53,56^, who reported that most groundwater samples were suitable for irrigation according to SAR classifications.

Noticeable improvements were observed in the RSC, where 61.54%, 46.15%, and 84.62% of the winter, spring, and summer samples, respectively, were classified as good (RSC < 1.25 meq/L), indicating reduced carbonate hazards. Similarly, most of the treated samples fell within the permissible Na% category (40–60%), while the KR classified 53.85%, 53.85%, 38.46%, and 38.46% of the winter, spring, summer, and autumn samples, respectively, as suitable. These findings agree with those of^52^ and^53^, who emphasized the importance of evaluating multiple irrigation indices simultaneously, as groundwater suitability may vary depending on the selected criterion.

All treated samples remained within acceptable PI classes, which is consistent with the findings of^56^ and^58^. Furthermore, substantial improvements were observed in the MH, PS and TH, indicating reduced magnesium-related hazards and water hardness after treatment. The CAI1 and CAI2 indices also revealed a gradual shift from reverse to direct ion exchange processes after treatment, indicating changes in groundwater hydrochemistry, which is consistent with observations reported in^58^.

Overall, a comparison with previous groundwater studies confirms that the AgNP/AC filtration system effectively reduced alkalinity, hardness, and magnesium-related irrigation hazards.

**Table 11:** Groundwater suitability for irrigation based on index guidelines after treatment.

Irrigation Indices	Recommended range	Categories	Wet winter	Dry spring	Dry summer	Wet autumn
			Percentage suitability of samples			
SAR	< 10	Excellent	100.00%	100.00%	100.00%	100.00%
	10–18	Good	---	---	---	---
	18–26	Doubtfully	---	---	---	---
	> 26	Unsuitable	---	---	---	---
RSC	< 1.25	Good	61.54%	46.15%	84.62%	---
	1.25–2.5	Doubtfully	15.38%	7.69%	15.38%	23.08%
	> 2.5	Unsuitable	23.08%	46.15%	---	76.92%
% Na	< 20	Excellent	---	---	---	---
	20–40	Good	7.69%	30.77%	15.38%	15.38%
	40–60	Permissible	84.62%	61.54%	84.62%	84.62%
	60–80	Doubtfully	7.69%	7.69%	---	---
	> 80	Unsuitable	---	---	---	---
KR	< 1	Suitable	53.85%	53.85%	38.46%	38.46%
	> 1	Unsuitable	46.15%	46.15%	61.54%	61.54%
PI	> 75%	Suitable	---	---	---	100.00%
	25–75%	Good	100.00%	100.00%	100.00%	---
	< 25%	Unsuitable	---	---	---	---
MH	< 50	Suitable	53.85%	61.54%	92.31%	100.00%
	> 50	Unsuitable	46.15%	38.46%	7.69%	---
PS	< 3	Suitable	100%	100%	100%	100%
	> 3	Unsuitable	---	---	---	---
CAI1	−ve	Reverse Exchange Process	84.62%	61.54%	53.85%	23.08%
	+ve	Direct Exchange Process	15.38%	38.46%	46.15%	76.92%
CAI2	−ve	Reverse Exchange Process	84.62%	61.54%	53.85%	23.08%
	+ve	Direct Exchange Process	15.38%	38.46%	46.15%	76.92%
TH	< 75	Soft	100.00%	76.92%	76.92%	100.00%
	75–150	Moderate	---	23.08%	23.08%	---
	150–300	Hard	---	---	---	---
	> 300	Very Hard	---	---	---	---

b) **Comparison with groundwater treatment studies employing activated carbon-based composite filtration systems**:

To further evaluate the effectiveness of the proposed AgNP/AC filtration system, the obtained results were compared with those of related nanocomposite, activated-carbon, and conventional treatment studies^77–79^ and are presented in Table 12. It must nevertheless be emphasized that no single previously published study constitutes an exact match for a benchmark, since the cited works differ in source–water type, baseline salinity, treatment medium, energy source, and operational scale. These studies nonetheless remain suitable for comparison, as each shares one or more key dimensions with the present system in terms of treatment material, source-water category, or reuse application.

The comparison shows that^77^ is relevant from the activated-carbon treatment perspective, as it compares ion exchange and activated carbon for improving well-water quality, with EC removal ranging from 36% to 73% and an average reduction of approximately 63%. However, that study did not report improvements in TDS or SAR, did not include microbiological assessment, and did not address renewable-energy operation or a pilot reuse framework. Therefore^77^, support the effectiveness of activated carbon as an adsorptive medium, but it does not provide a complete comparison with the present AgNP/AC system.

In contrast, the study in^78^ is the closest comparison to the present study in terms of treatment material, as it uses silver nanoparticles impregnated on activated carbon in a continuous packed-column system. Its main strength lies in providing sustained antibacterial performance, with complete *E. coli* removal under continuous-flow operation and monitoring of silver release in the treated water. However^78^, was performed using model *E. coli*-contaminated Milli-Q water rather than real groundwater, and its objective was drinking-water disinfection rather than irrigation reuse. Additionally, the work did not evaluate salinity, TDS, SAR, or irrigation suitability. Accordingly^78^, confirmed the antimicrobial value of AgNPs/AC, whereas the present study expands this concept to real shallow coastal groundwater and combines microbial reduction with physico-chemical improvement and verification of irrigation suitability.

Among the compared studies^79^, provides the closest application-based benchmark, as it treated saline groundwater for agricultural reuse using a modified activated carbon/bentonite composite. The reported EC decreased from 2.54 to 1.12 dS/m, corresponding to an approximately 56% reduction, whereas the TDS decreased from 1440 to 716.8 mg/L, corresponding to an approximately 50% reduction. SAR also decreased from 5.3 to 2.7, and the irrigation classification improved from C4–S2 to C3–S1. In comparison, the present AgNP/AC system achieved equal or greater relative improvements, reducing the EC by approximately 70–80%, the TDS by approximately 60–75%, and the SAR to values mostly below 2. Consequently, most treated samples in the present study fell within the “Excellent” irrigation suitability category.

Overall, the comparison indicates that the proposed system combines advantages that were separately addressed in previous studies. Like^77^, it benefits from activated-carbon adsorption; similar to^78^, it incorporates silver-based antimicrobial functionality, and similar to^79^, it targets groundwater treatment for irrigation reuse. However, unlike these studies, the present work integrates physico-chemical improvement, microbiological assessment, irrigation suitability verification, continuous seasonal monitoring, and solar-supported operation within a single campus-scale treatment-and-reuse framework. This integrated configuration represents the main distinction of the proposed AgNP/AC filtration system.

**Table 12:** Comparison of the proposed AgNP/AC filtration system with previously reported activated carbon-based groundwater treatment systems.

Parameter	^77^	^78^	^79^	Present Study
Main similarity to present study	Uses activated carbon for well-water treatment	Uses AgNPs supported on activated carbon	Treats groundwater for irrigation reuse	Integrates activated carbon adsorption, AgNPs disinfection, and irrigation reuse
Treatment material	Activated carbon; ion exchange	Ag-NPs on plasma-treated activated carbon (Ag-AC)	Fe-modified activated carbon/bentonite	Ag-NPs/AC
Water type (region)	Groundwater wells (Kufa, Iraq)	Model *E. coli* in Milli-Q water (India)	Saline groundwater (Sohag, Egypt)	Shallow coastal groundwater (Abu Qir, Alexandria, Egypt)
Application & scale	Irrigation/domestic; lab treatment, 2 seasons	Drinking-water disinfection; lab continuous-flow column	Irrigation reuse; + 2-season crop trial	Irrigation reuse; campus-scale pilot, continuous seasonal monitoring
Energy source	Conventional	Conventional	Conventional	RES
EC	Average 63% (ranging 36–73%) ion exchange ↓41%	NR	2.54 → 1.12 dS/m (56%)	~ 0.66–1.06 → ~0.14–0.26 dS/m (ranging ≈ 70–80%, average 54.2%)
TDS	NR	NR	1440 → 716.8 mg/L (50%)	~ 330–608 → ~85–157 mg/L (ranging ≈ 60–75%, average 47.3%)
SAR	NR	NR	5.3 → 2.7	~ 2.66–3.43 → mostly < 2
Microbiological assessment	Not evaluated	High *E. coli* inactivation (continuous)	Not evaluated	Evaluated (TPC, TAC, TC, FC)

NR: not reported; N/A = not applicable.

### Limitations and future work

While the proposed AgNP/AC filtration system clearly improved groundwater physicochemical quality, some limitations should be acknowledged. Most salinity-related parameters, major ions, and nutrient indicators decreased after treatment and remained within the adopted irrigation guidelines; however, not all parameters were fully controlled. In particular, Cu²⁺ remained above the adopted FAO limit even in the pre-treatment dataset, suggesting possible copper contamination in the study area. Likewise, although NO₂^-^ and acidity were markedly reduced (by approximately 47.8% and 58.3%, respectively), their post-treatment averages (1.81 mg/L for NO₂^-^ and 12.63 mg/L for acidity) remained slightly above the adopted FAO limits of 1 mg/L and 10 mg/L, and turbidity frequently exceeded the permissible limit, particularly during spring and summer. These findings indicate that, although the system improved the overall physicochemical profile of the groundwater, an additional filtration or polishing stage is required for the parameters that remained above guideline values.

Microbiological performance showed similar limitations. Elevated TPC values remained detectable in some samples after treatment, particularly during spring and summer. Although total and fecal coliform concentrations stayed below the detection limit, satisfying the commonly adopted FAO and WHO irrigation guidelines for fecal indicator bacteria, the persistence of TPC indicates that the system did not achieve complete microbial disinfection, so a complementary disinfection step would be needed where stricter microbiological standards apply.

Beyond these treated-water observations, several aspects were outside the scope of the present study and define the priorities for future work. The long-term behaviour of the filtration medium under continuous field operation, including breakthrough behaviour, adsorption-capacity exhaustion, regeneration efficiency, and filter lifespan, was not characterized and should be established to support scale-up. In parallel, silver nanoparticle leaching, dissolved silver concentrations in the treated water, and long-term silver accumulation in irrigated soils were not measured; although the AgNPs were immobilized on activated carbon, gradual silver release during prolonged operation cannot be excluded. Accordingly, future work should focus on quantifying leaching as well as assessing composite stability when subject to repeated filtration and regeneration cycles. Moreover, possible effects on soil microbial communities, crop yields and environmental sustainability need to be addressed. Finally, further studies on ion removal, enhancement of salinity hazard reduction, and integration with other desalination or disinfection technologies, together with large-scale field trials and long-term soil impact assessments are suggested to ascertain the practical feasibility, scalability, and reliability of the system for sustainable agricultural water reuse.

## Conclusion

The growing water scarcity in Egypt indicates the need for the sustainable management and reuse of groundwater as a complementary resource for irrigation. In this paper, an integrated framework for the seasonal monitoring, irrigation suitability and solar-assisted abstraction of groundwater is proposed using an AgNP/AC nanocomposite filtration technique, which is experimentally evaluated under real operating conditions at the AASTMT campus in Abu Qir, Alexandria, Egypt. The results revealed that the groundwater quality was seasonally variable, especially during spring and summer, with higher salinity, turbidity, dissolved ions, and microbial contamination than in autumn and winter. The AgNP/AC filtration system showed a significant improvement in the various physical, chemical and hydrochemical parameters after treatment. In particular, there were significant reductions in salinity related parameters, major dissolved ions, hardness, residual sodium carbonate and possible salinity. The irrigation suitability indices were also improved overall after treatment, indicating reduced hazards of salinity, carbonate, hardness, and magnesium, while the SAR values remained in the excellent irrigation class. The main contribution of this work lies in moving beyond conventional groundwater assessment toward an integrated, application-oriented reuse framework. Unlike most previous studies in Egypt, which focused mainly on groundwater diagnosis, hydrochemical characterization, suitability classification, or health-risk evaluation, the present study combines monitoring, treatment, renewable-energy operation, and reuse assessment in a single field-based system. The proposed campus-scale pilot framework integrates groundwater abstraction, treatment, and irrigation reuse, which remains limited in the Egyptian groundwater literature. Nevertheless, the findings also indicate that further optimization is required before wider application. Although the system improved the overall groundwater quality, some parameters, especially turbidity, microbial indicators and selected irrigation-related indices of some samples, were not consistently within the limits of the adopted guidelines after treatment. Future work should therefore focus on long-term continuous operation, filter regeneration and breakthrough behavior, Ag release and leaching stability, microbial safety under extended field conditions, technoeconomic assessment, scalability and life cycle evaluation. These investigations are critical for confirming the long-term reliability, economic feasibility and practical applicability of the proposed solar-supported AgNP/AC filtration system for decentralized groundwater reuse.

## Data Availability

Data are contained within the article.
